# Supplementary material for: Supporting evidence-based decisions about the geographic and demographic extensions of seasonal malaria chemoprevention in Benin: A modelling study
Source: PLOS Glob Public Health. 2025 May 19;5(5):e0004509. doi: 10.1371/journal.pgph.0004509 (PMC12088001; doi:10.1371/journal.pgph.0004509)
Supplement: S1 Appendix — Figure A2: Proportion of population in each age group in Benin according to 2013 census. Figure A3: Monthly incidence by department averaged between 2011 and 2017. Figure A4: ITN use assumptions by department. Figure A5: Type of nets distributed or planned to be distributed in 2020, 2023 and 2026. Figure A6: Assumptions for ingredients and population coverage of IRS campaigns between 2011 and 2021. Figure A7: Case management cascade from access to care measured in 2017–2018 Demographic and Health Survey at the national level. Figure A8: Evolution of access to care (blue) and effective treatment coverage (green) by department. Figure A9: SMC implementation in children under 5 between 2019 and 2023. Figure A10: Calibrated simulations for communes of Alibori. Figure A11: Calibrated simulations for communes of Atacora. Figure A12: Calibrated simulations for communes of Atlantique. Figure A13: Calibrated simulations for communes of Borgou. Figure A14: Calibrated simulations for communes of Collines. Figure A15: Calibrated simulations for communes of Couffo. Figure A16: Calibrated simulations for communes of Donga. Figure A17: Calibrated simulations for Cotonou, commune of Littoral. Figure A18: Calibrated simulations for communes of Mono. Figure A19: Calibrated simulations for communes of Ouémé. Figure A20: Calibrated simulations for communes of Plateau. Figure A21: Calibrated simulations for communes of Zou. Figure B1: Percentage of reduction of all predicted malaria episodes or severe cases induced by SMC demographic (A) or geographic (B) extension between 2024 and 2026 by age group. Figure B2: Absolute averted malaria episodes, severe cases and deaths by each extension scenario. (DOCX) [file pgph.0004509.s001.docx]

**Supporting evidence-based decisions about the geographic and demographic extensions of seasonal malaria chemoprevention in Benin: a modelling study - Supplementary material**

Jeanne Lemant, Clara Champagne, William Houndjo, Julien Aïssan, Rock Aïkpon, Camille Houetohossou, Sakariahou Kpanou, Roland Goers, Cyriaque Affoukou, Emilie Pothin

Contents

[**A.** **Calibration workflow** 1](#_Toc187424740)

[Country data 1](#_Toc187424741)

[Calibration fits 7](#_Toc187424742)

[**B.** **Additional results** 12](#_Toc187424759)

[Effect sizes 12](#_Toc187424760)

[Averted cases 13](#_Toc187424761)

[**C.** **References** 14](#_Toc187424762)

## **Calibration workflow**

### Country data

#### Population


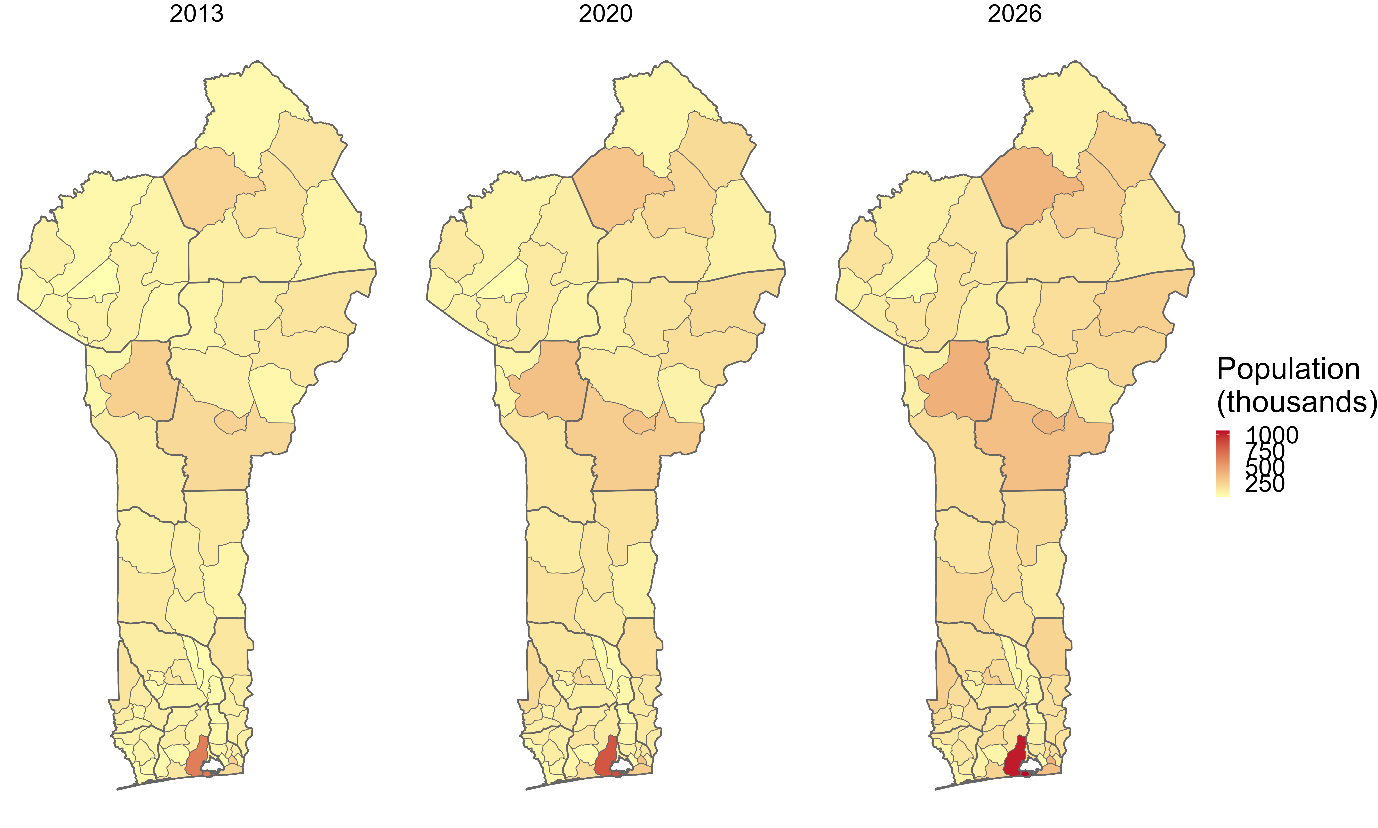


**Figure A1: Population by commune in 2013, 2020 and 2026.**

The population was estimated from the 2013 census [1] at the commune level, applying a 3∙51% annual growth rate to each commune (national growth rate between 2002 and 2013 censuses). Base layer of Benin map from <https://data.humdata.org/dataset/cod-ab-ben>.

Age structure

**
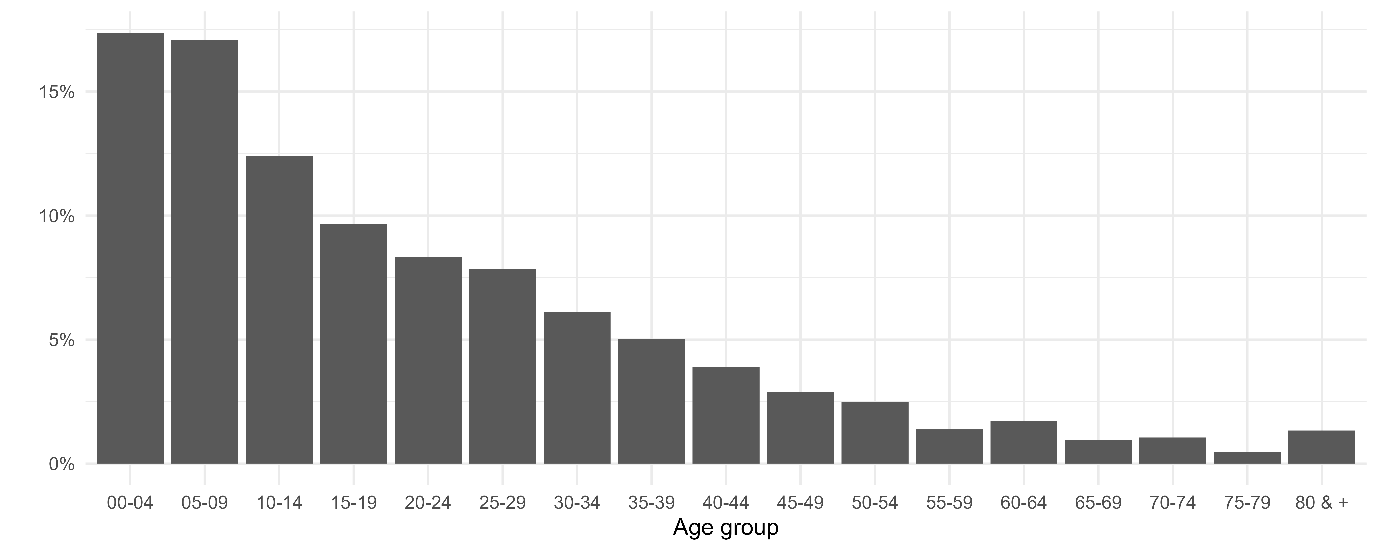
**

**Figure A2: Proportion of population in each age group in Benin according to 2013 census [1].**

#### Seasonality


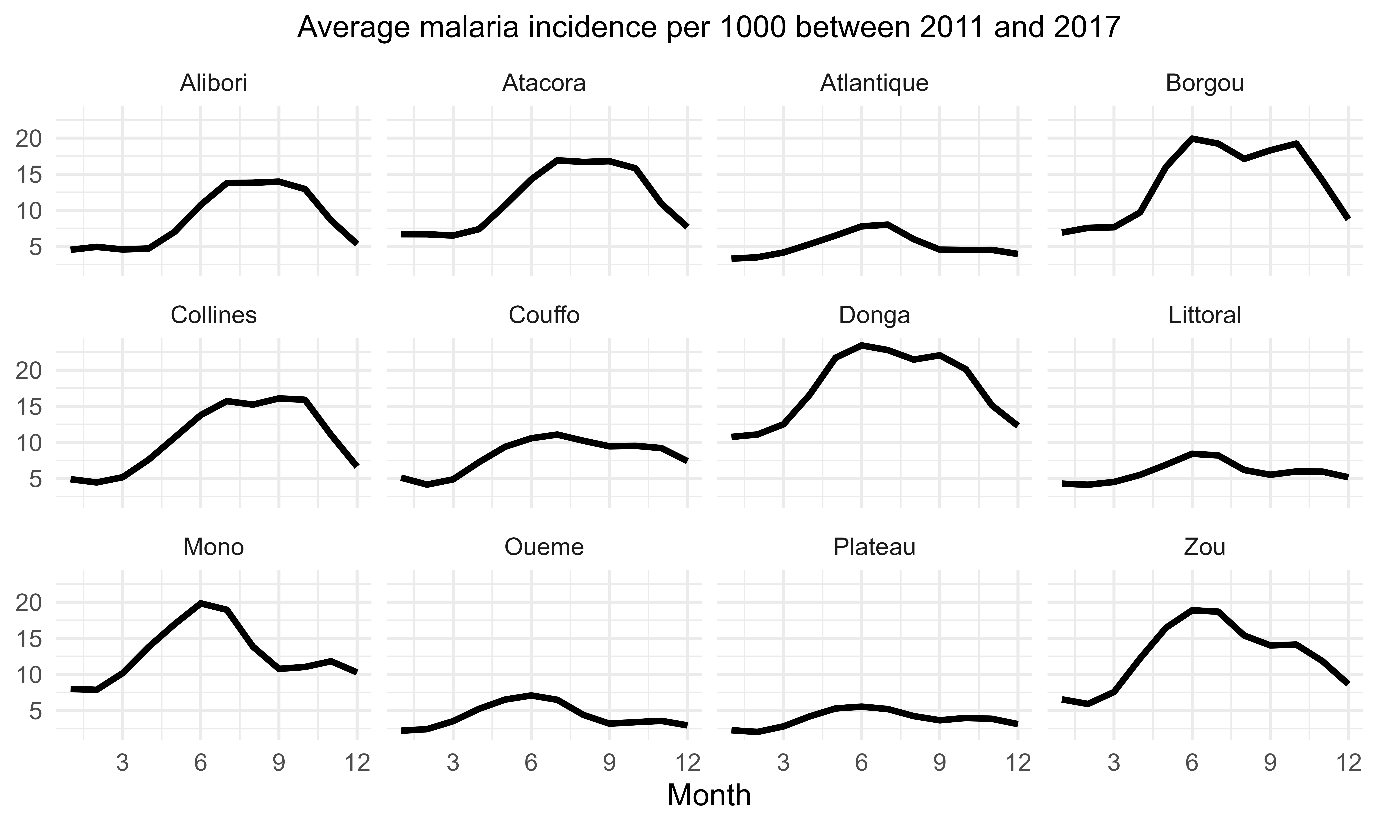


**Figure A3: Monthly incidence by department averaged between 2011 and 2017.**

Reported incidence was calculated using monthly DHIS2 confirmed cases averaged between 2012 and 2017 and population estimates from 2013 census.

#### Insecticide-Treated Nets


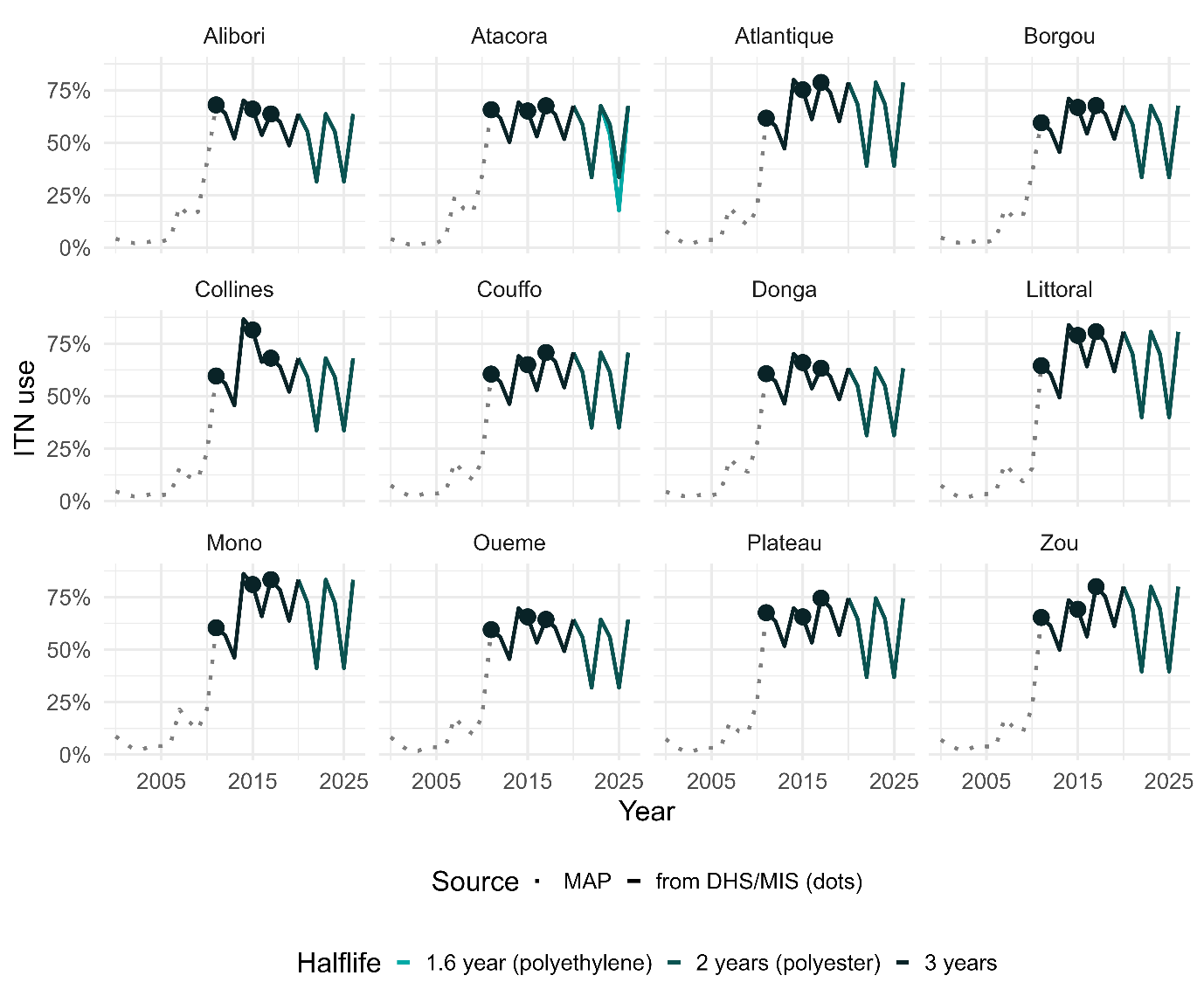


**Figure A4: ITN use assumptions by department.**

Until 2011 we use the yearly estimates from the Malaria Atlas Project, from 2011 to 2019 we use surveys, and assume nets have a three-year halflife in-between distributions. From 2020 we kept the initial use as measured after the 2017 campaign but adjusted the halflife to the fabric of distributed nets. In 2023 two types of nets (polyester and polyethylene) were distributed in Atacora, and we modelled the correct type in each commune of the department.


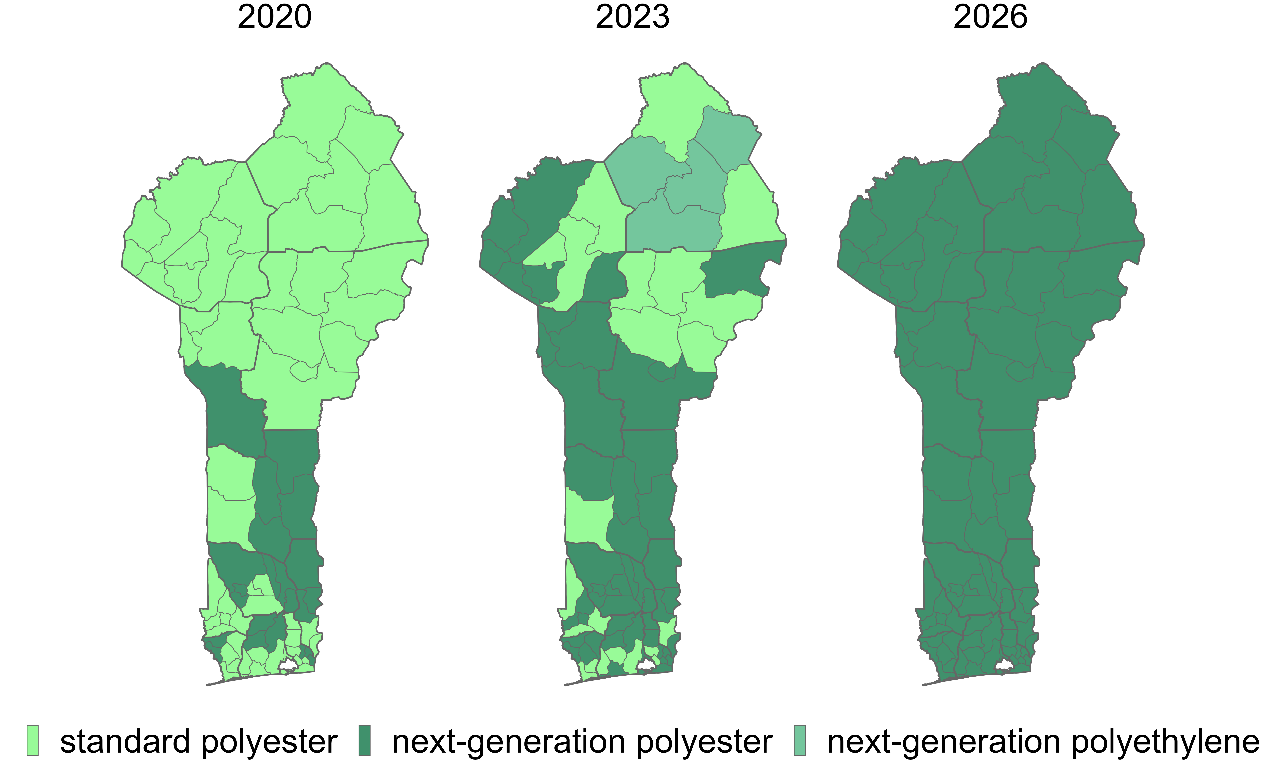


**Figure A5: Type of nets distributed or planned to be distributed in 2020, 2023 and 2026.**

Base layer of Benin map from <https://data.humdata.org/dataset/cod-ab-ben>.

#### IRS


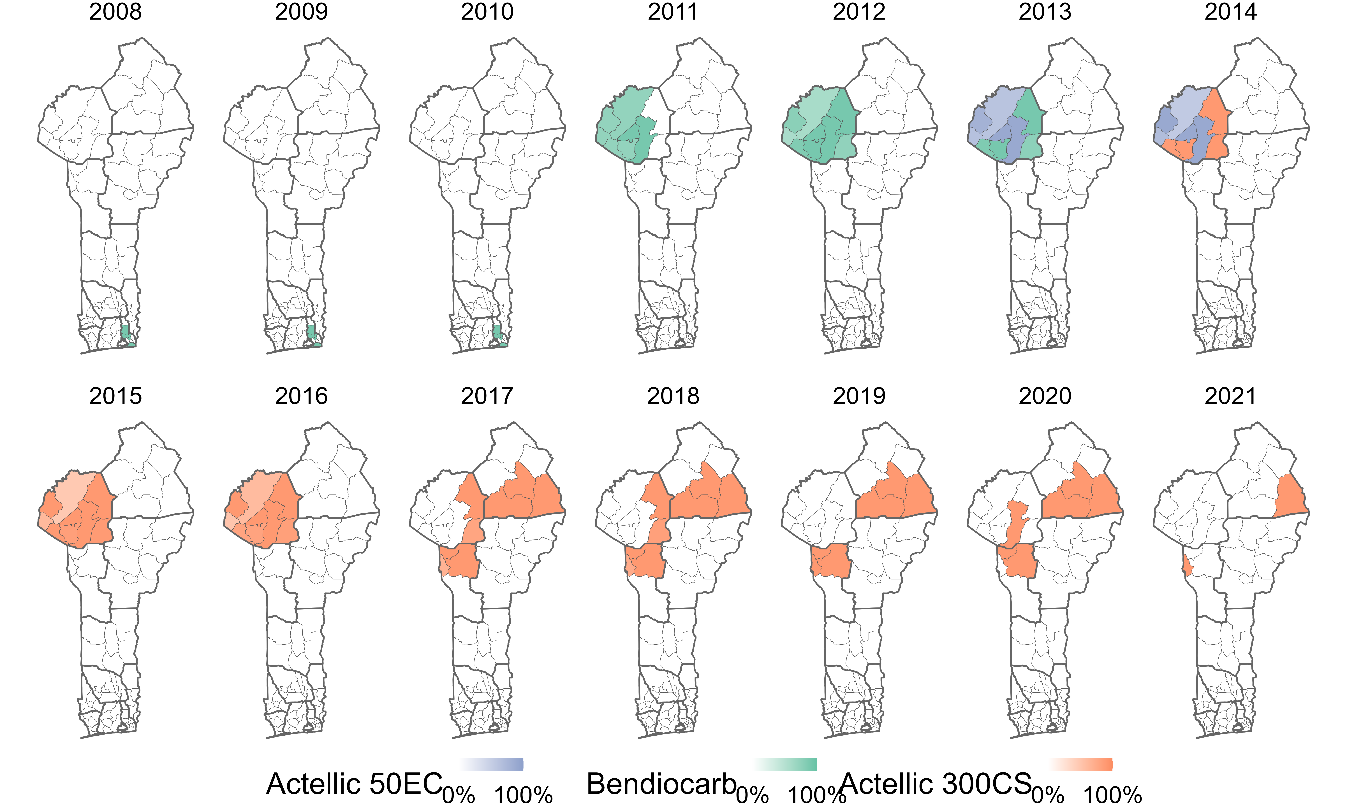


**Figure A6: Assumptions for ingredients and population coverage of IRS campaigns between 2011 and 2021.**

In 2020 and 2021 Pirimiphos-methyl was sprayed but the most recent parameterisation available was for Actellic 300CS. Base layer of Benin map from <https://data.humdata.org/dataset/cod-ab-ben>.

#### Case management


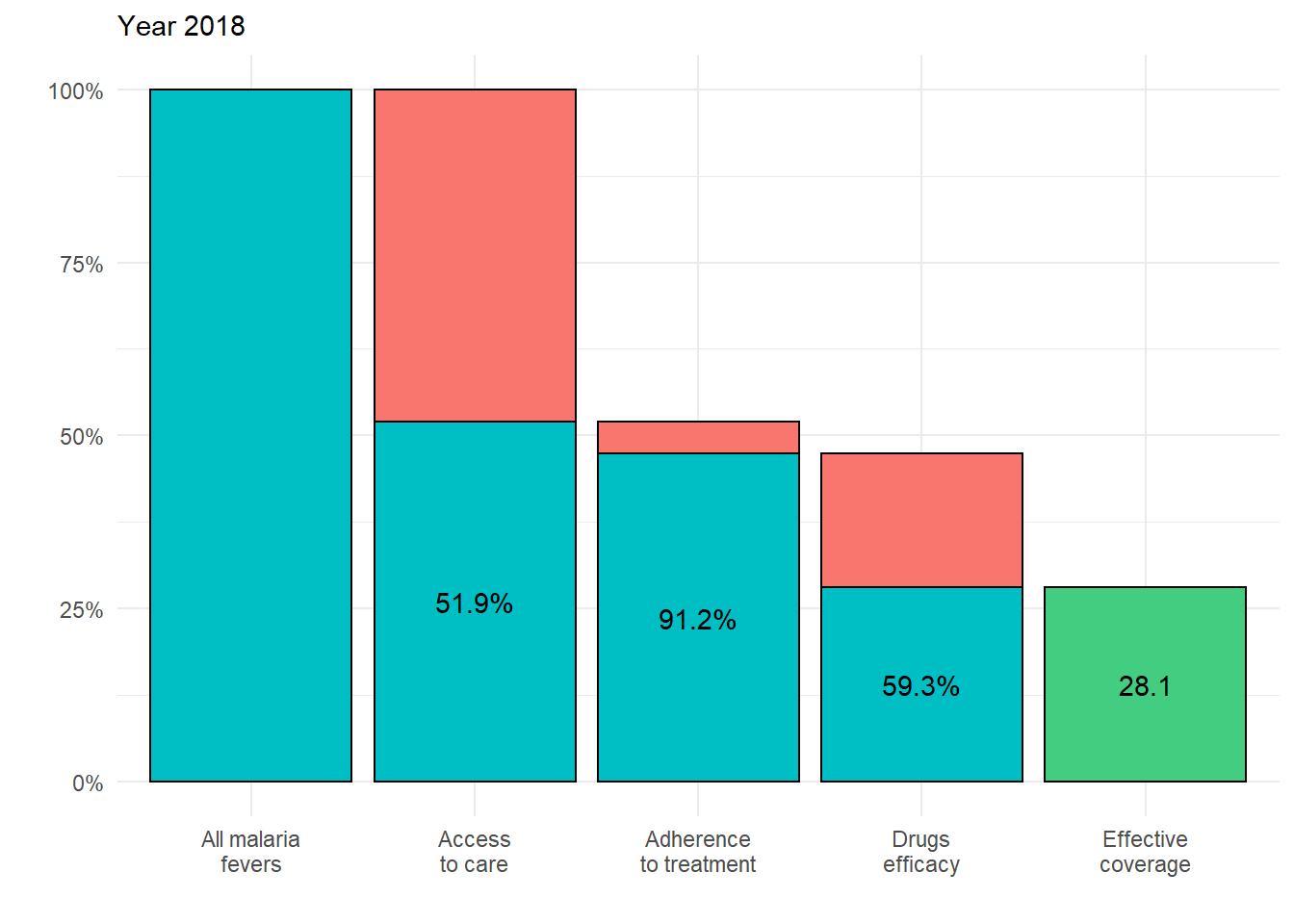


**Figure A7: Case management cascade from access to care measured in 2017-2018 Demographic and Health Survey [2] at the national level.**


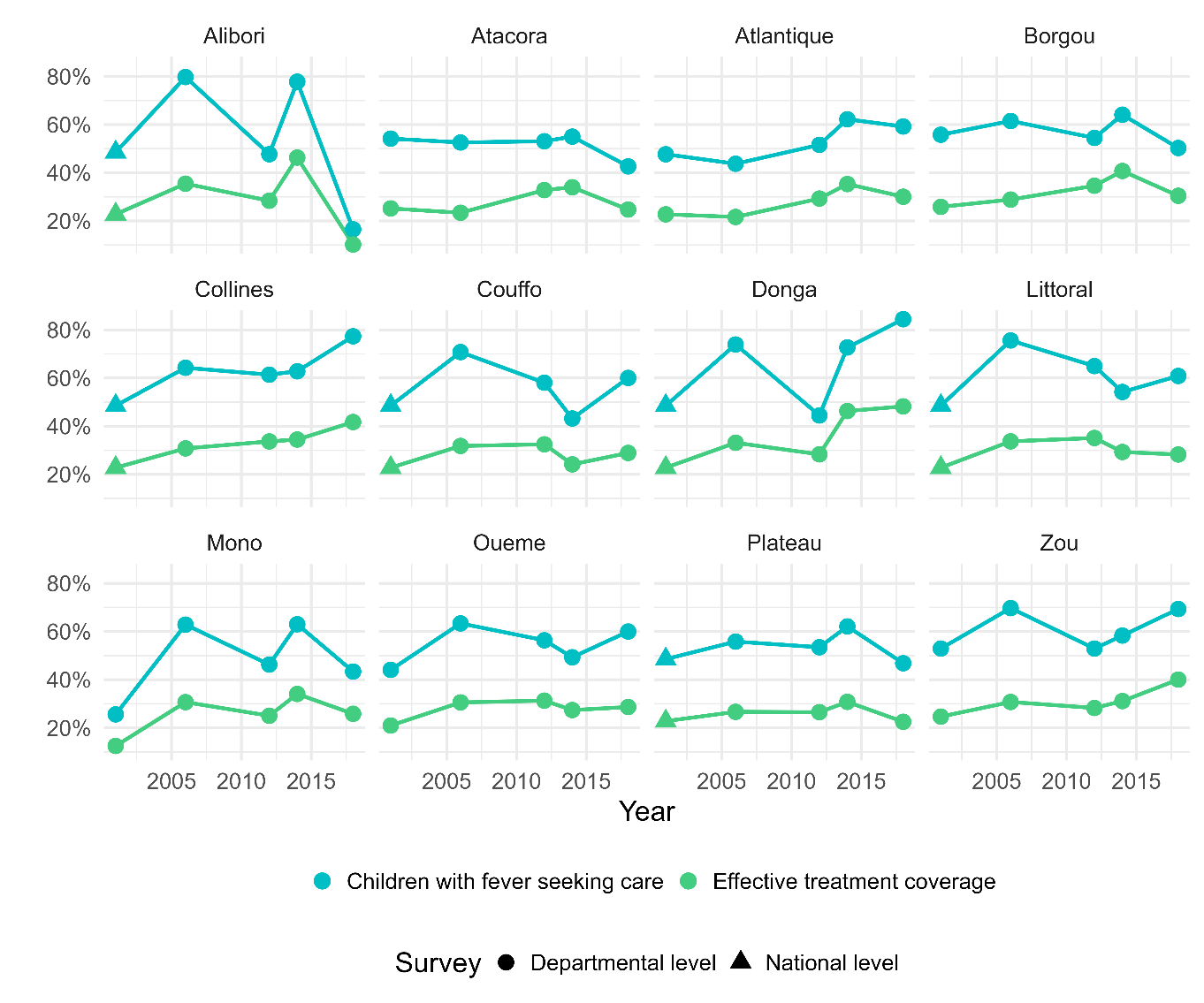


**Figure A8: Evolution of access to care (blue) and effective treatment coverage (green) by department.**

#### SMC


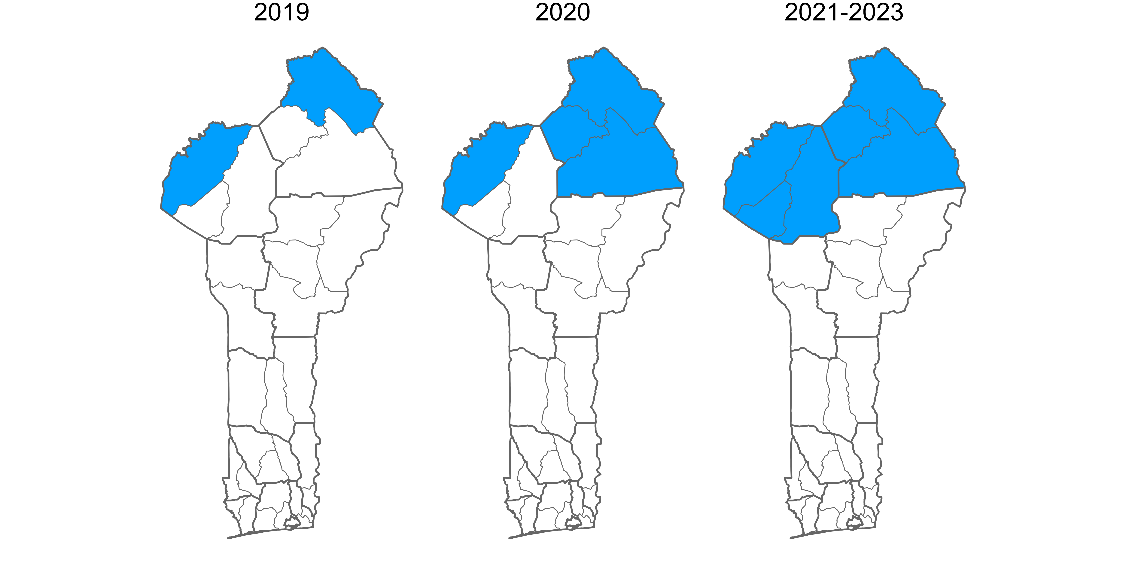


**Figure A9: SMC implementation in children under 5 between 2019 and 2023.**

Base layer of Benin map from <https://data.humdata.org/dataset/cod-ab-ben>.

### Calibration fits

For each department, we show commune-level prevalence estimates from the Malaria Atlas Project in children from 2 to 10 (in green) and calibrated simulations for each commune. Individual simulation runs are represented in black as well as estimated uncertainty (in grey).


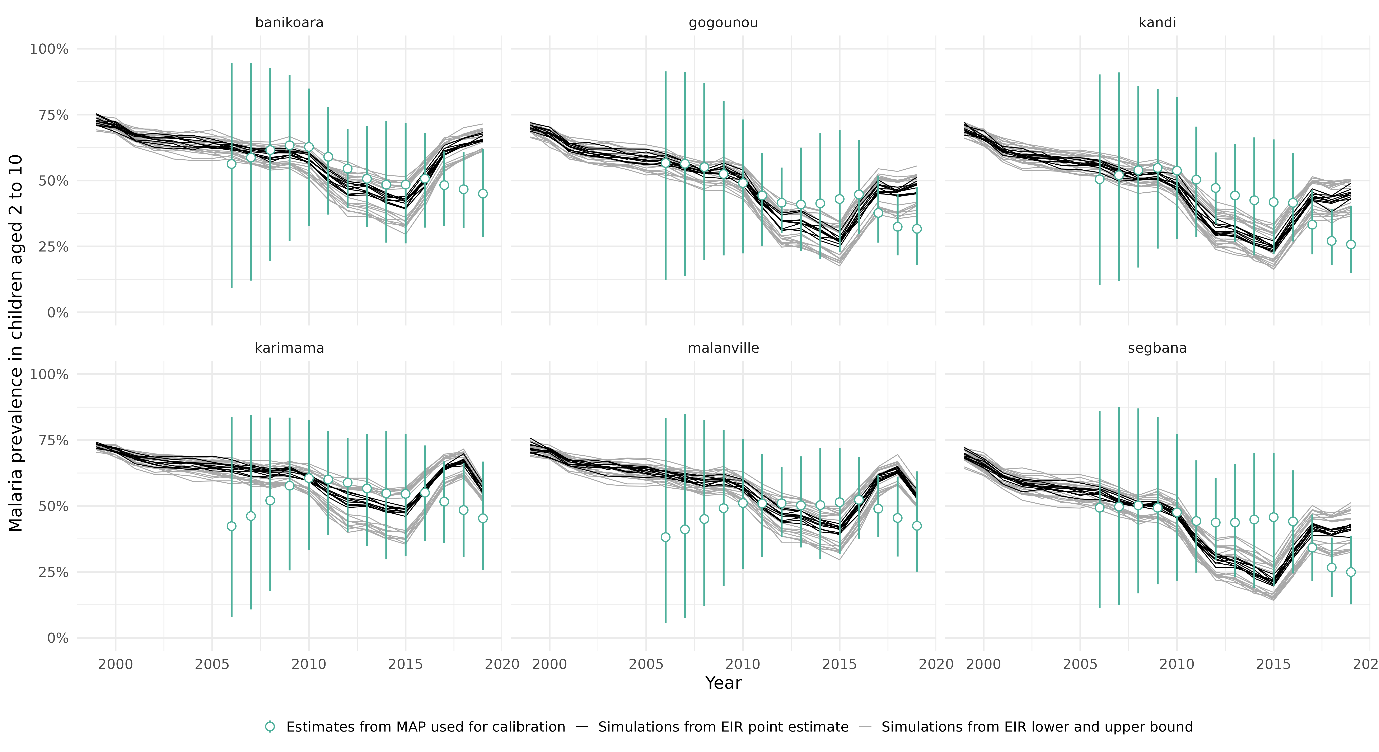


**Figure A10: Calibrated simulations for communes of Alibori.**


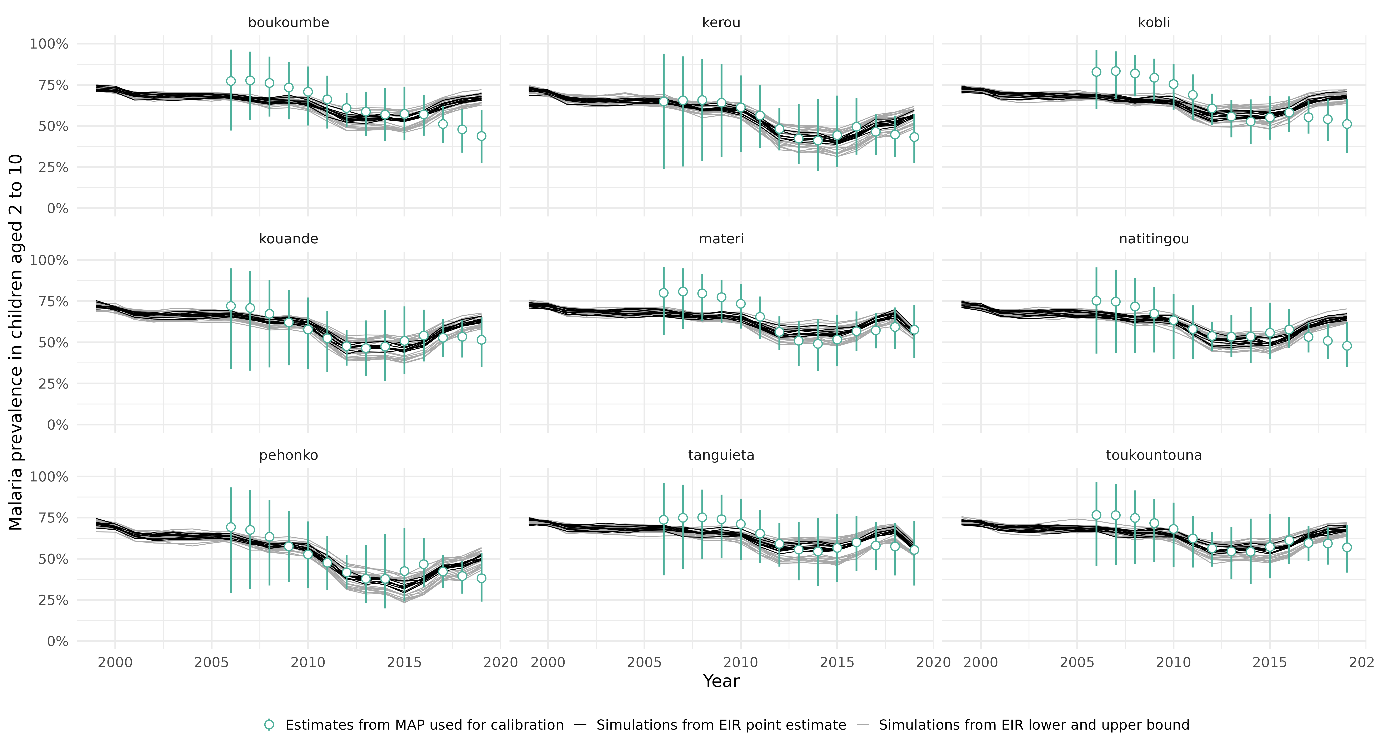


**Figure A11: Calibrated simulations for communes of Atacora.**


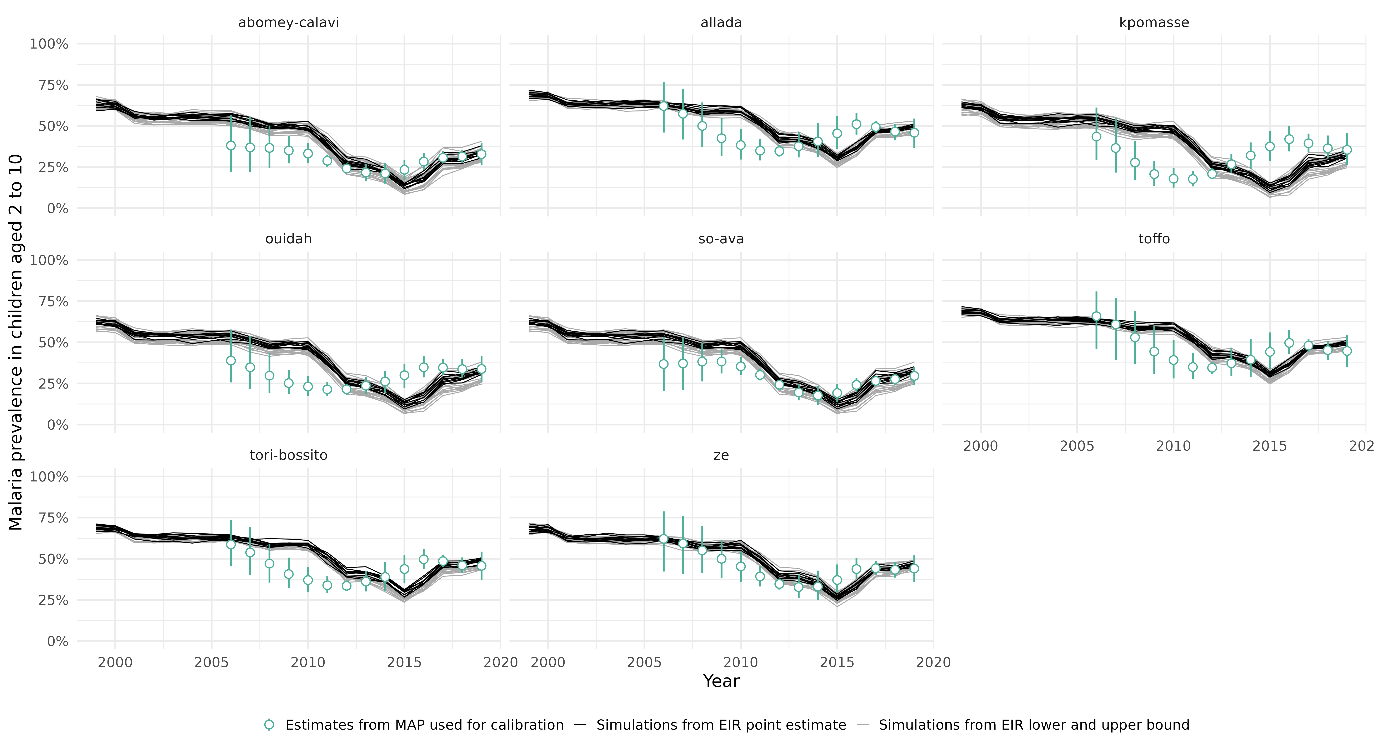


**Figure A12: Calibrated simulations for communes of Atlantique.**


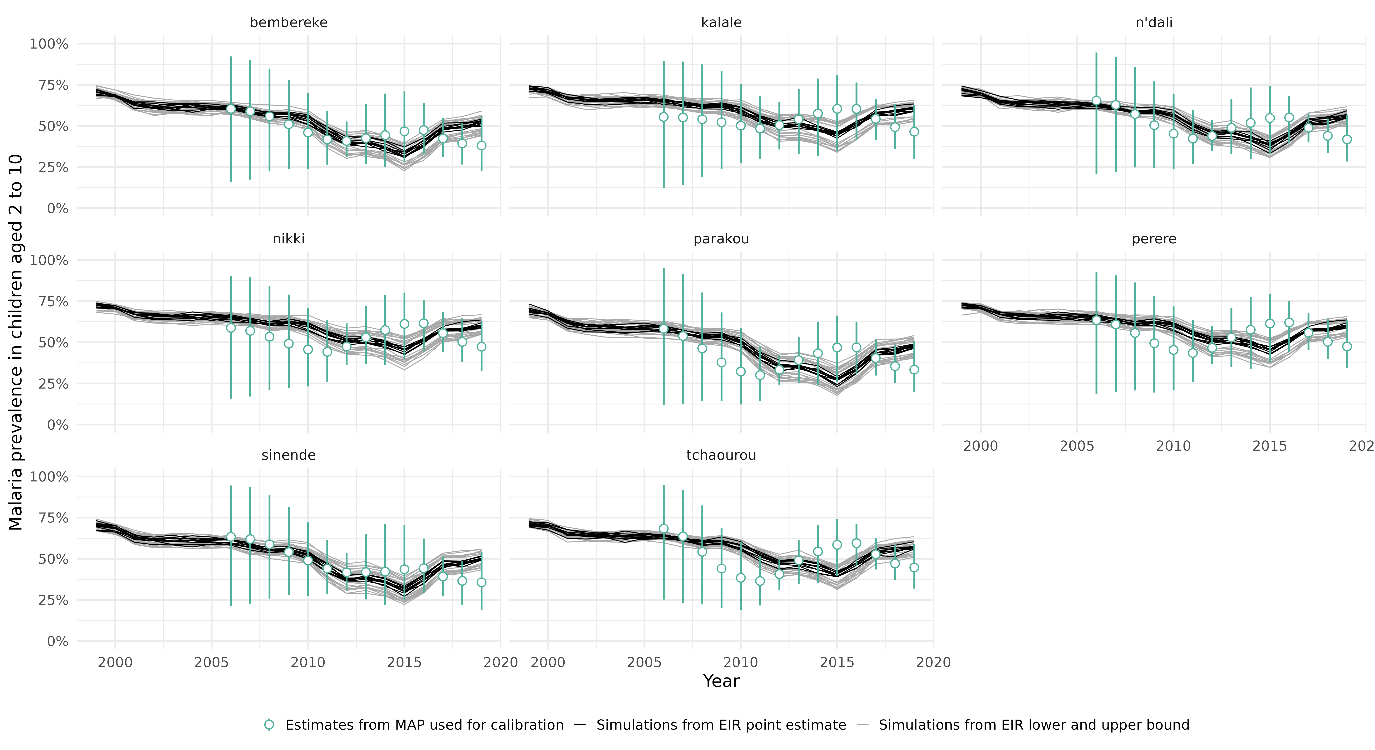


**Figure A13: Calibrated simulations for communes of Borgou.**


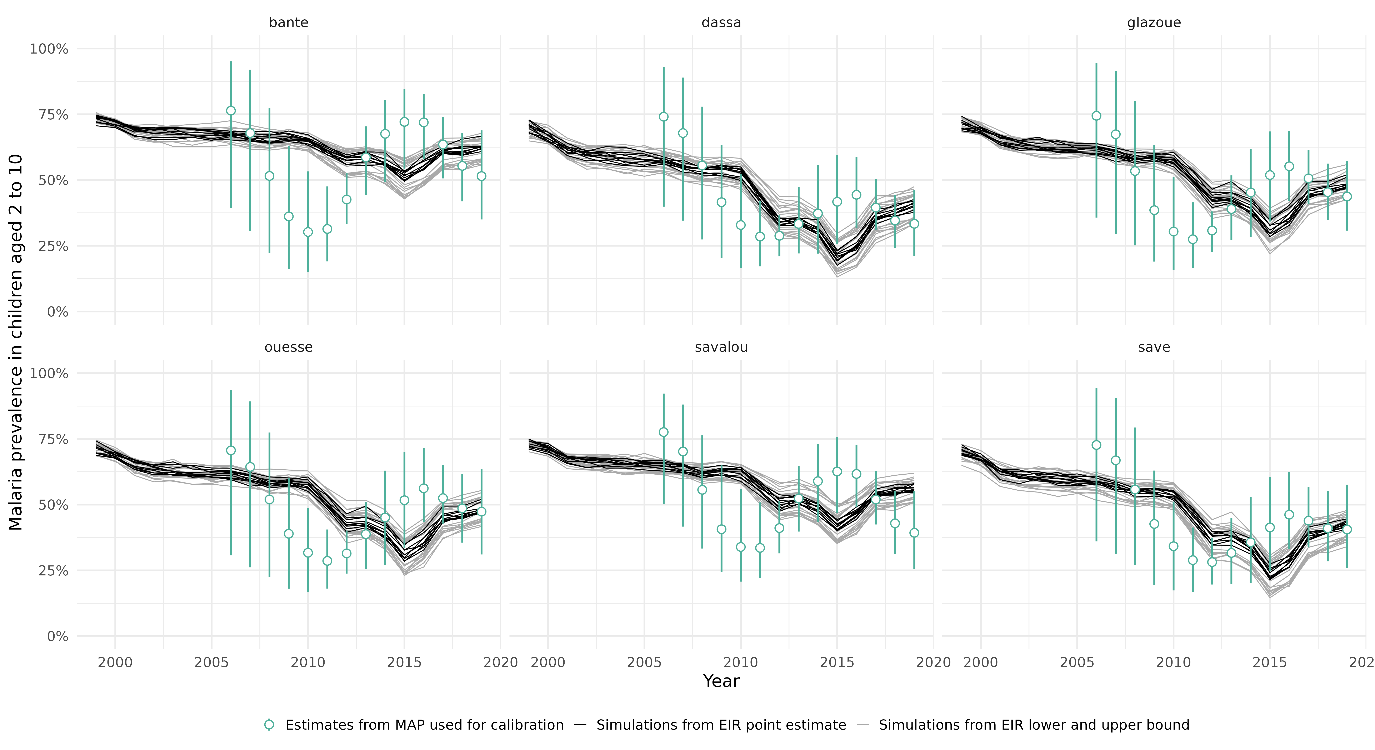


**Figure A14: Calibrated simulations for communes of Collines.**


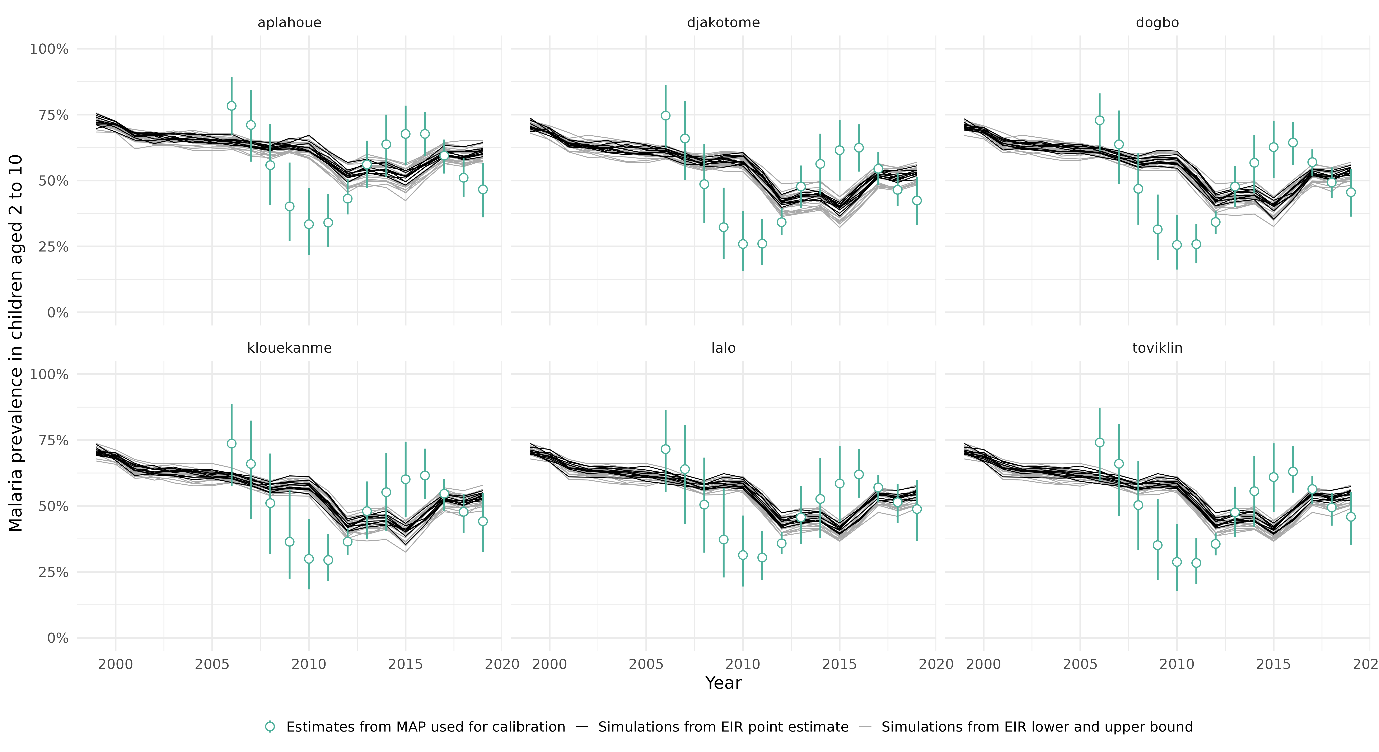


**Figure A15: Calibrated simulations for communes of Couffo.**


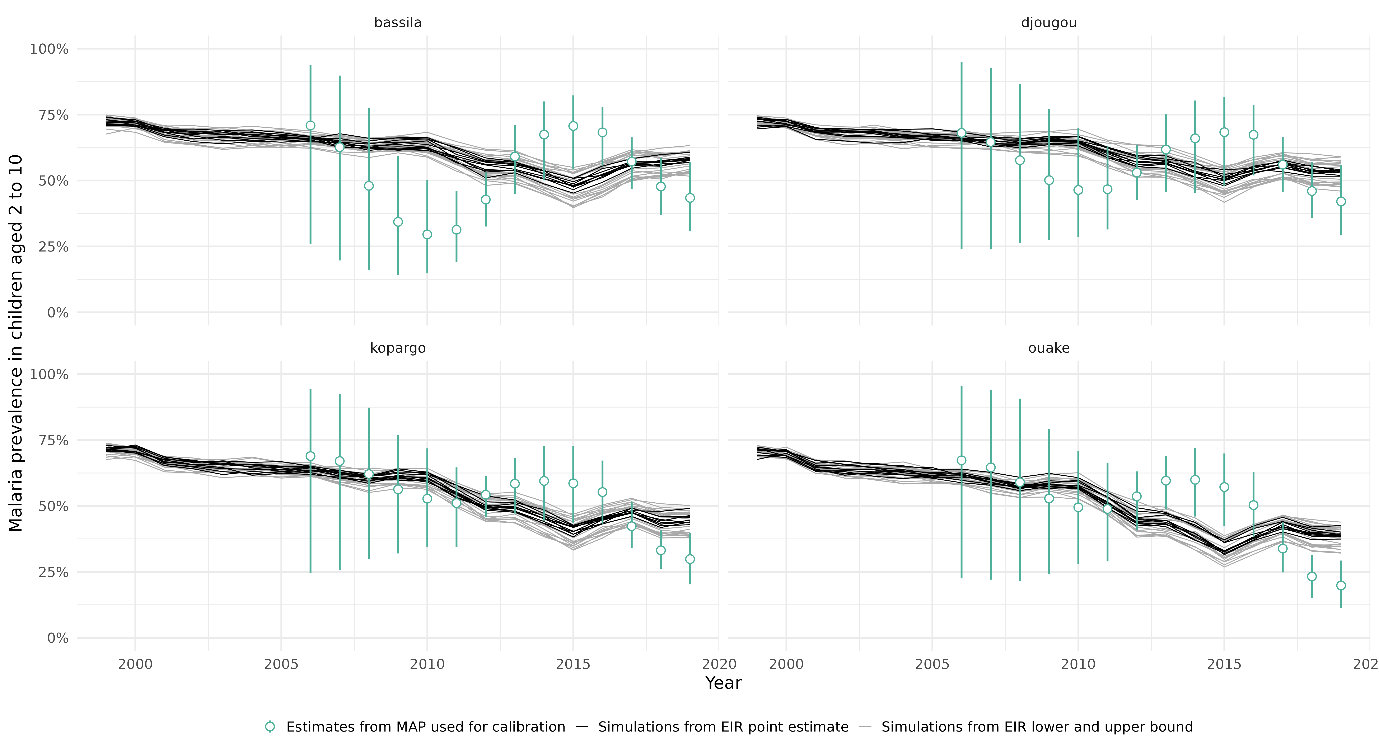


**Figure A16: Calibrated simulations for communes of Donga.**


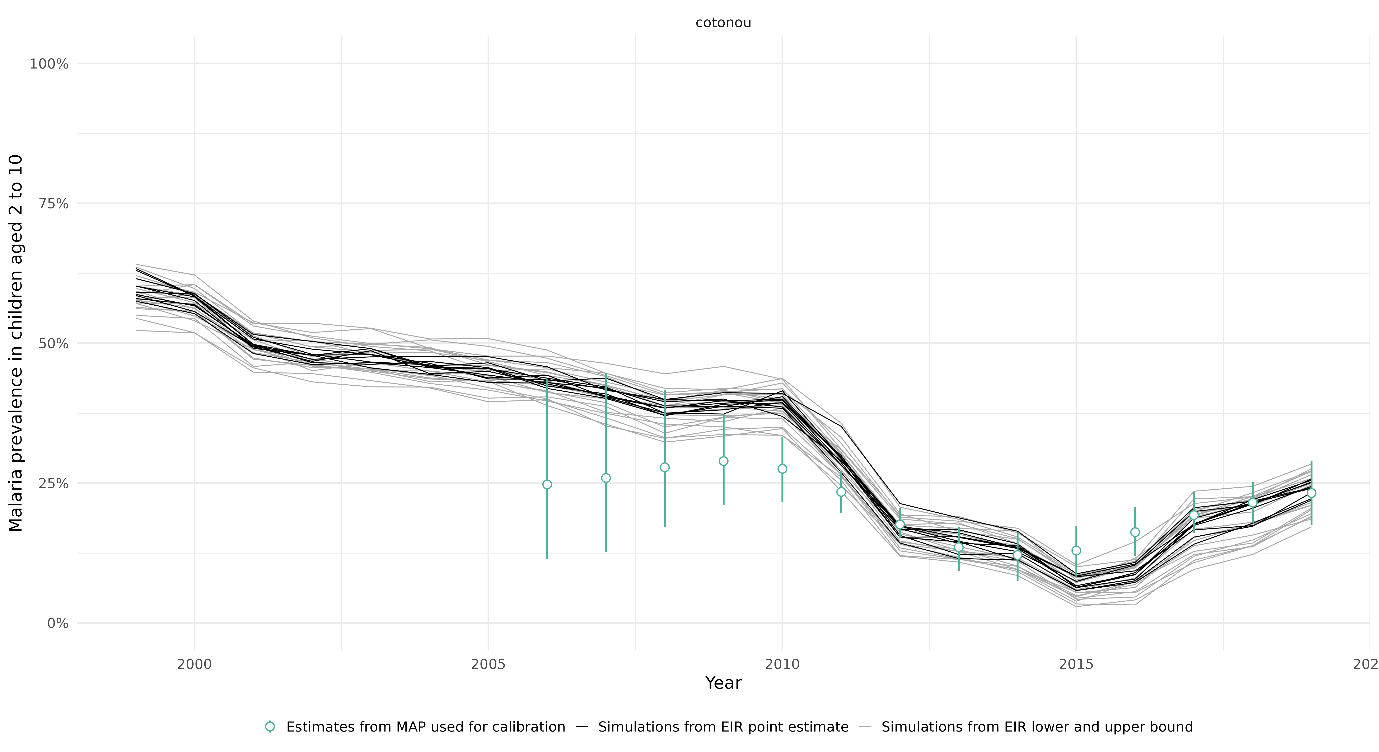


**Figure A17: Calibrated simulations for Cotonou, commune of Littoral.**


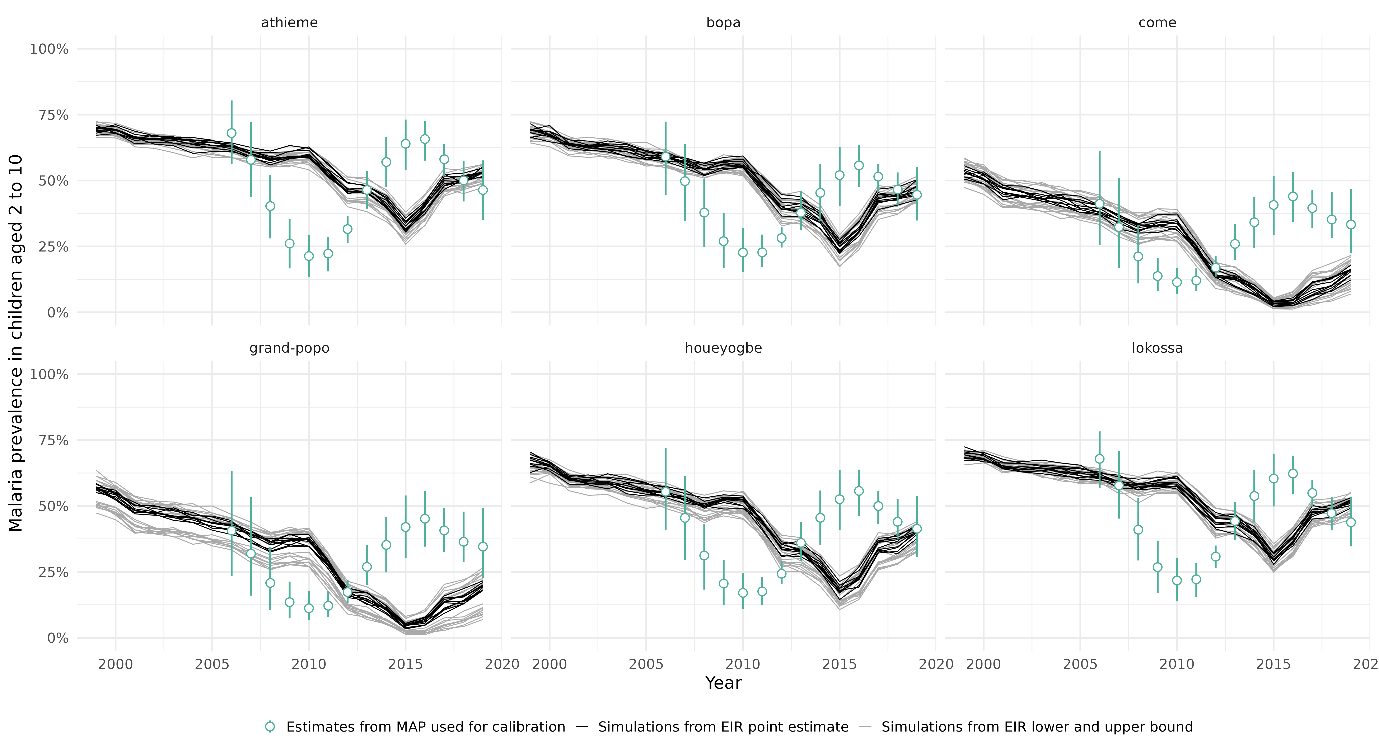


**Figure A18: Calibrated simulations for communes of Mono.**


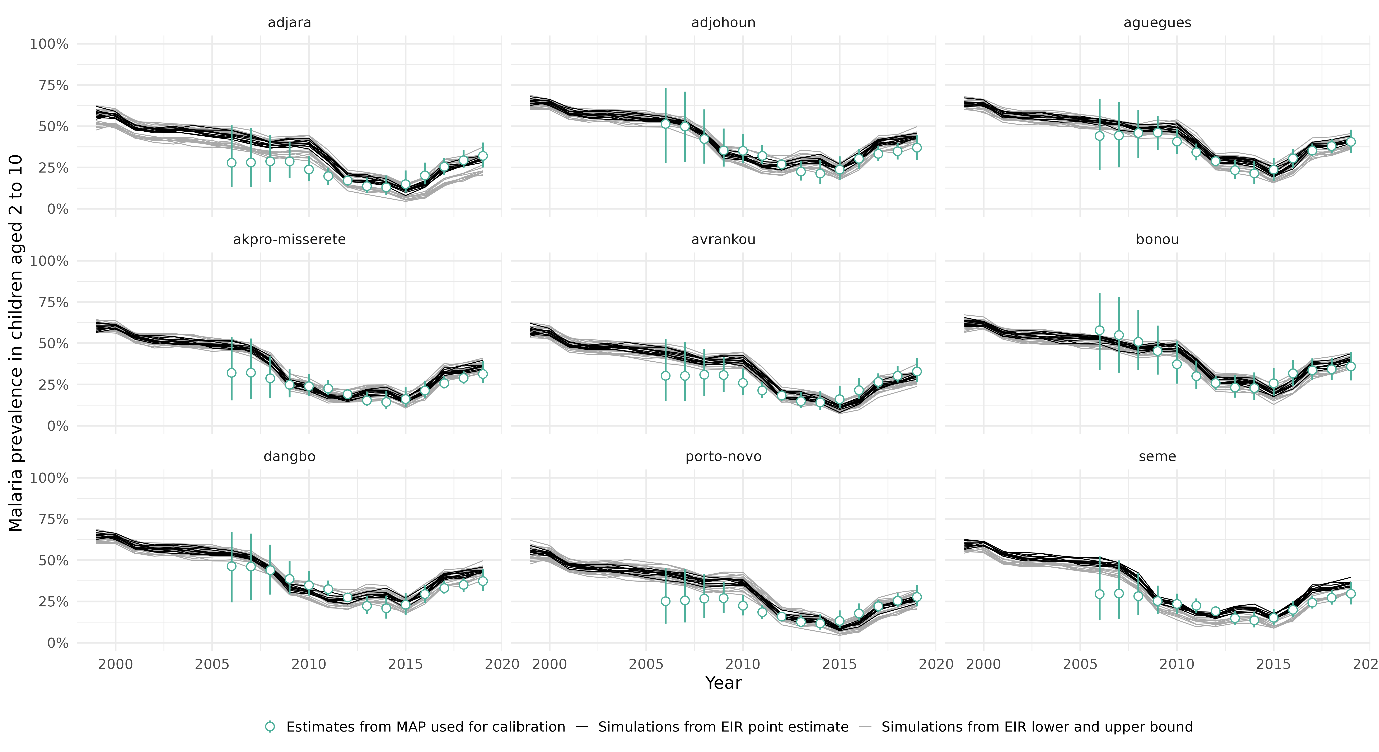


**Figure A19: Calibrated simulations for communes of Ouémé.**


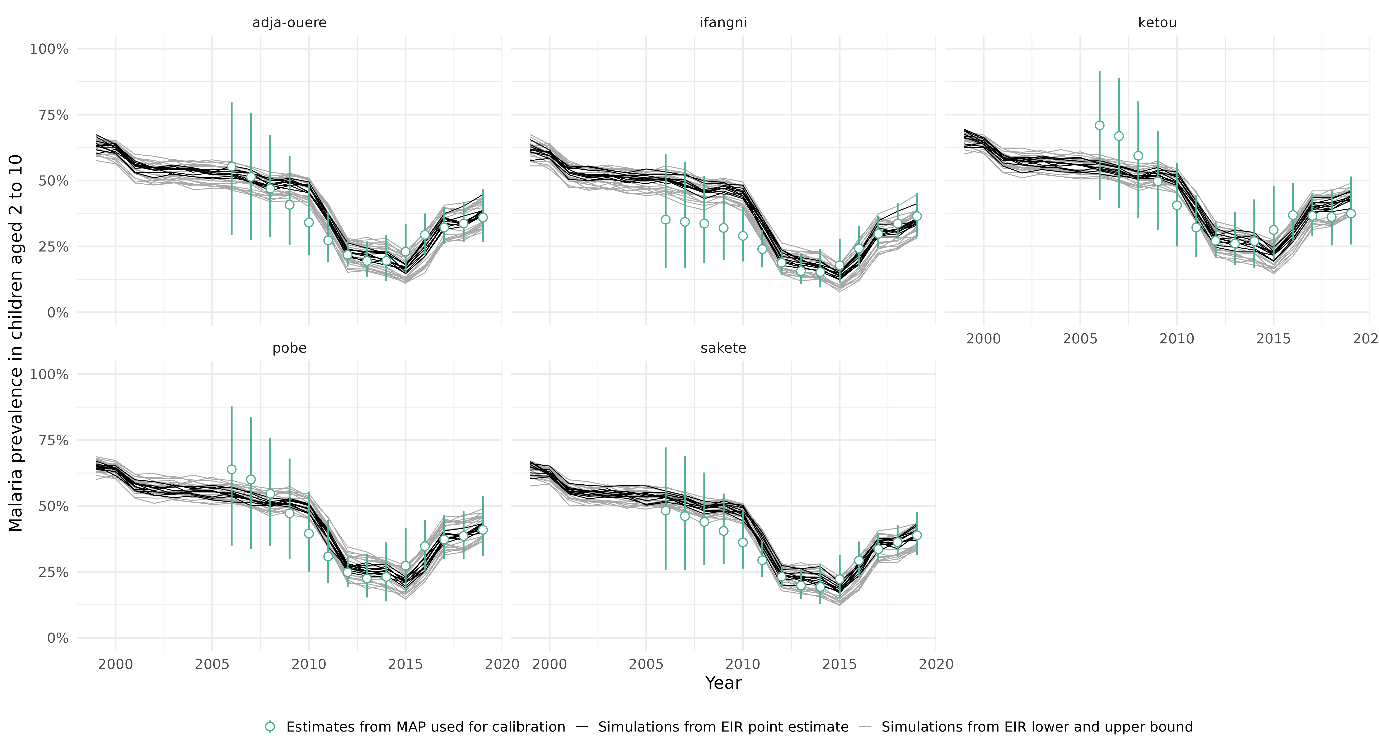


**Figure A20: Calibrated simulations for communes of Plateau.**

**
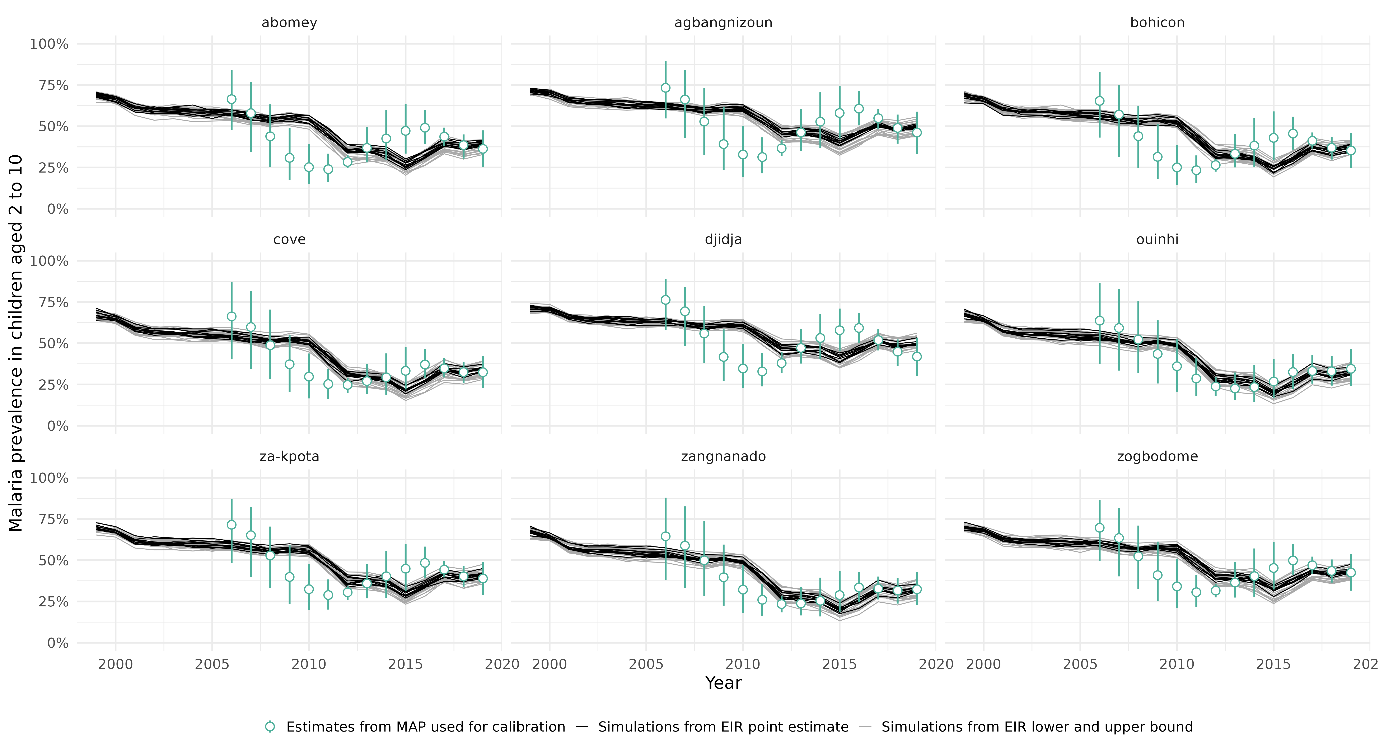
**

**Figure A21: Calibrated simulations for communes of Zou.**

## **Additional results**

### Effect sizes

We compare the reduction in malaria episodes and severe cases induced by both SMC extension scenarios compared to planned interventions only.

In Alibori and Atacora the model predicted 2.3 (2.1, 2.4) million malaria episodes in children from 5 to 10 years old between 2024 and 2026 with the planned interventions, and 1.7 (1.6, 1.7) with the demographic extension of SMC (see Figure 5 in main paper). This would represent a 26.2% (24.9, 26.9) reduction in malaria episodes and 25.5% (16.6, 29.7) in severe cases in children aged 5 to 10.


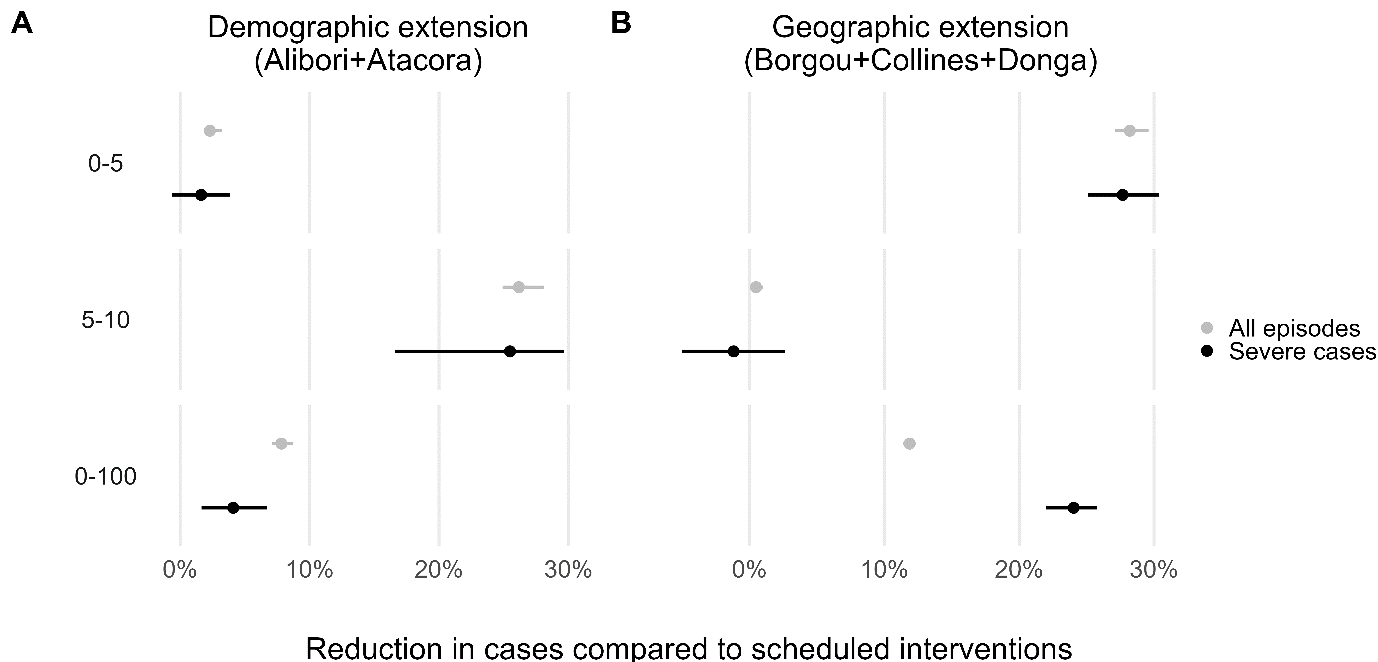


**Figure B1: Percentage of reduction of all predicted malaria episodes or severe cases induced by SMC demographic (A) or geographic (B) extension between 2024 and 2026 by age group.**

Error bars represent uncertainty on intensity of transmission as well as model stochasticity.

In zones eligible to the geographic extension, the model predicted 5.1 (4.6, 5.4) million malaria episodes in children under 5 between 2024 and 2026 under the planned interventions, against 3.7 (3.3, 3.9) million with the geographic extension of SMC. This would represent a 28.2% (27.1, 28.2) reduction in malaria episodes and 27.7% (25.1, 27.7) in severe cases in children under 5.

The impact of the demographic extension on the general population would be smaller than the one of the geographical extension, with malaria episodes in all ages diminishing by 7.8% (7.1, 7.8) and severe cases by 4.1% (1.7, 6.7) with the geographic extension compared to planned interventions, respectively 11.9% (11.4, 12.3) and 24% (22, 25.8) with the geographic extension.

### Averted cases


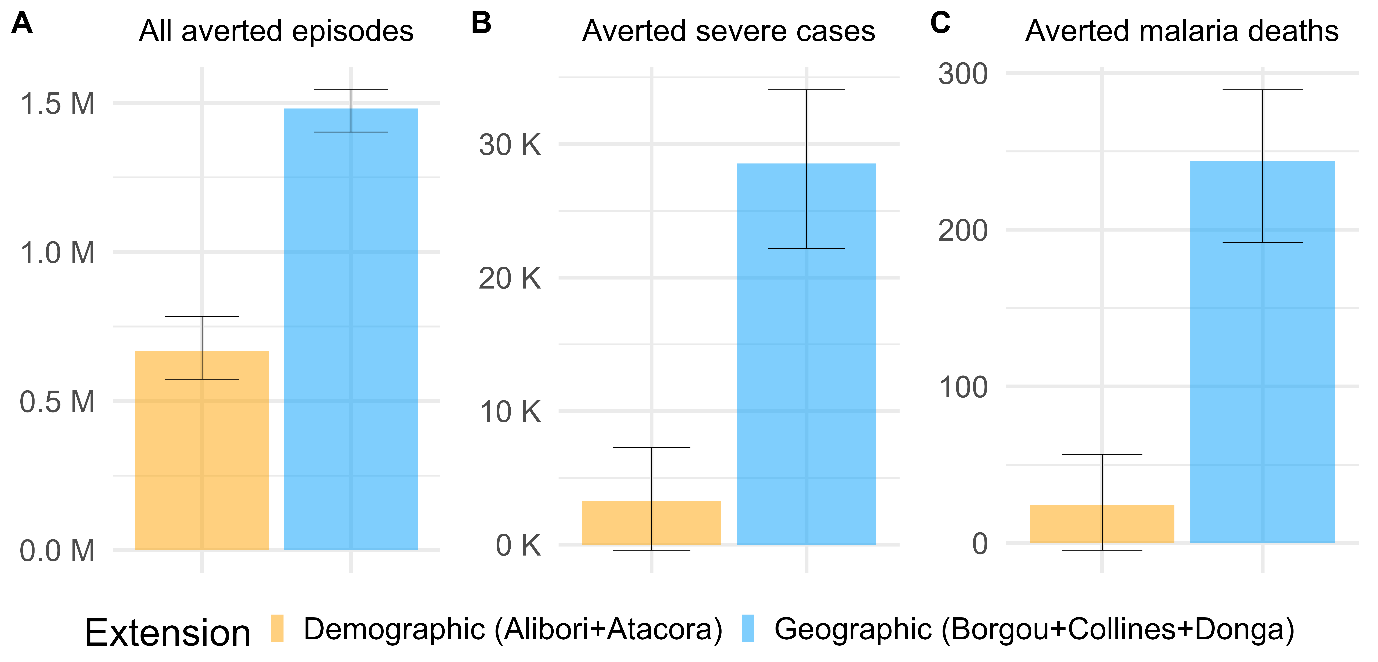
**Figure B2: Absolute averted malaria episodes, severe cases and deaths by each extension scenario.** A: All malaria episodes in all age groups averted by each SMC extension in eligible zones between 2024 and 2026; B: Severe cases averted between 2024 and 2026; C: Malaria deaths averted between 2024 and 2026. Error bars represent uncertainty on intensity of transmission as well as model stochasticity.

## **References**

1. Institut National de la Statistique et de l'Analyse Économique (INSAE). Quatrième Recensement Général de la Population et de l'Habitation (RGPH4) 2015 [Available from: <https://instad.bj/statistiques/statistiques-demographiques#population>.

2. Demographic and Health Surveys In Benin 2001-2018 [Available from: <https://dhsprogram.com/methodology/survey-search.cfm?pgtype=main&SrvyTp=country&ctry_id=52>.
