# Supplementary material for: Supporting evidence-based decisions about the geographic and demographic extensions of seasonal malaria chemoprevention in Benin: A modelling study
Source: PLOS Glob Public Health. 2025 May 19;5(5):e0004509. doi: 10.1371/journal.pgph.0004509 (PMC12088001; doi:10.1371/journal.pgph.0004509)
Supplement: Attachment — Submitted filename: Response to Reviewers.docx [file pgph.0004509.s003.docx]

January 28^th^, 2025

Dear Editor,

We thank the Reviewers for their thorough read of our work and their constructive comments, which greatly improved the manuscript. We have edited the article to address all the comments and provided a detailed answer to each of them below. All edits in the text are depicted in **bold** in the responses below.

We thank you very much for your time and consideration.

Sincerely,

Emilie Pothin, on behalf of all authors

**Journal Requirements:**

**1. Please note that PLOS ONE has specific guidelines on code sharing for submissions in which author-generated code underpins the findings in the manuscript. In these cases, we expect all author-generated code to be made available without restrictions upon publication of the work. Please review our guidelines at** [**https://journals.plos.org/plosone/s/materials-and-software-sharing#loc-sharing-code**](https://scanmail.trustwave.com/?c=22737&d=q8mh53_2hhc5A1Fnb5gRS4-7okSzHpRCIhWB-3cCKA&u=https%3a%2f%2fjournals%2eplos%2eorg%2fplosone%2fs%2fmaterials-and-software-sharing%23loc-sharing-code) **and ensure that your code is shared in a way that follows best practice and facilitates reproducibility and reuse.**

All the code for data manipulation, input parameters and processing used in the manuscript is now available on GitHub. The link, alongside those of the pre-existing code for the OpenMalaria mathematical model and its user workflow is also now in the updated the Data Availability statement:

***Model code can be found here: https://github.com/SwissTPH/openmalaria and here: https://github.com/SwissTPH/r-openMalariaUtilities. Code for data manipulation, input parameters and processing is available here: https://github.com/SwissTPH/BeninSMC.***

**Review Comments to the Author
Reviewer #1: This a paper modelling seasonal Malaria Chemoprevention to compare treating 0-5 children in previously untreated areas vs extending SMC to 5-10 in previously treated areas.
 The authors conclude that geographical extension is likely to prevent more cases than demographic extension.

 The research appears to be sound, they are using a "OpenMalaria" model, but I could not see the code. I believe this would be important if others want to apply the same kind of analysis.**

Thank you for this remark. We have now provided all the code used in the manuscript on GitHub and updated the Data Availability statement as follows:

***Model code can be found here: https://github.com/SwissTPH/openmalaria and here: https://github.com/SwissTPH/r-openMalariaUtilities. Code for data manipulation, input parameters and processing is available here: https://github.com/SwissTPH/BeninSMC.***

**The article focuses on a particular situation (Benin) and is essentially a report geared at the health authorities.
 It is argued that this is relevant since the comparison builds on many parameters and could change depending on the particular environment.
 However, this makes it difficult to understand why the conclusion leaned towards a particular choice, and then what could be extrapolated to other locations, other populations.**

Thank you for this important comment.

Although our analysis is specific to Benin, the question of choosing between a geographical and demographical extension of SMC is very likely to be asked in many other malaria endemic countries.

There is currently no straightforward answer to this question: children under 5 years old are more at risk of severe disease while older children are a large parasitic reservoir and overall, burden shifts to older age groups as the endemicity level decreases (as shown in Uganda in Kigozi et al. 2020, <https://pmc.ncbi.nlm.nih.gov/articles/PMC7106889/>). Therefore, the way these effects outweigh each other is very likely to be highly context-specific. In our case, what is driving the result is the high proportion of severe cases in children under 5 due to the high transmission intensity in all the departements considered for the SMC extension scenarios. However, another yet unpublished analysis being conducted in our group to compare SMC extensions in different countries found that the optimal strategy depended on the geographies, their transmission intensities and the other interventions already deployed. Overall, it is very likely that there is not a one size-fits-all answer, and that each country needs to makes specific choices based on their own data.

This is in line with several WHO initiatives (High Burden to High Impact [https://www.who.int/publications/i/item/WHO-CDS-GMP-2018.25 page 4](https://www.who.int/publications/i/item/WHO-CDS-GMP-2018.25%20page%204) or sub-national tailoring <https://cdn.who.int/media/docs/default-source/malaria/mpac-documentation/mpag-march2024-session4-subnational-tailoring-of-interventions-rev.pdf?sfvrsn=6eeebf97_3>) which advocate for using more country-specific data to make decisions. The WHO recommendation on SMC is now that “Malaria programmes should assess the suitability of SMC based on the local malaria epidemiology and available funding” and our manuscript is therefore an illustration of how such assessments can be conducted in a novel way using evidence from mathematical modelling to complement their current understanding.

In order to include your feedback and better reflect these considerations, we have updated the manuscript as follows:

We rewrote the introduction to explain why the question of how to extend SMC is relevant to many countries, not just Benin, and what the pros and cons of each approach could be:

***Most severe malaria cases and deaths occur among children under 5 especially under high transmission intensities [12-14], which calls for a geographic extension of SMC to protect those most at risk. But older children can represent a large parasitic reservoir [15] and as young children are protected, burden may shift to school-aged children [16]. Several countries have already adopted interventions targeting school-aged children, such as Intermittent Preventive Treatment in Tanzania [17] or SMC in children under 10 in Mali [18]. Moreover, the impact of SMC in different age groups may be influenced by factors such as intensity of transmission, seasonality or the concomitant deployment of other interventions, which vary both across and within countries. Therefore, it is not straightforward to anticipate which SMC strategy would be most impactful in a given context, and setting-specific analyses using the available local data are required, as encouraged by the WHO [5, 19].***

We have also developped a section in the discussion to highlight what is likely the main driver of the conclusion obtained in Benin :

***The superiority of the geographic extension scenario in northern Benin is most likely due to the over-representation of children under 5 among severe cases in high transmission settings. In OpenMalaria*** *severe malaria episodes can result from high parasitaemia and/or* ***co-occurrence of risk factors, with*** *the latter increasing with EIR and strongly decreasing with age [45].* ***In all of Benin’s departments under consideration for SMC extensions,*** *malaria risk is high: we therefore estimated high EIR values* ***during our calibration (up to 176 infective bites per person per year in Tanguieta, Atacora) and*** *this explains why our results attribute around 90% of severe cases to children under 5.* ***This is in line with several studies which found that at high transmission intensities, young children bear most of the burden of severe cases [12-14]. Since the WHO recommends to target SMC to “children belonging to age groups at high risk of severe malaria” [4], we considered severe cases as one of our main indicators, which is why geographically extending SMC to more children under 5 was found to be more beneficial – but it may not be so in areas with different transmission intensities, or with other interventions already targeting young children.***

**At the same time, the study is not really a "how to" aiming at making the same computation in other situations.**

Thank you for this remark. In order to ensure our work is reproducible by others, the code is now public (<https://github.com/SwissTPH/BeninSMC>) and we have substantially expanded the Methods section. In particular, we have provided additional details on model parameters, calibration methodology and results, and scenarios definition and comparison as explained in the answers below.

Additionally, we hope that publishing our work will give more visibility to such initiatives bringing together malaria programmes and mathematical modellers to support decision-making at the country level and that it could encourage others to do so.

**There are a number of parameters that are fixed to default values due to lack of data.**

Thank you for this comment. The parameters that are fixed to default values are taken from previous calibration work on OpenMalaria and they mosly correspond to the general biology of malaria, which we consider not to be specific to Benin. We believe that we used local data whenever available and relevant to the differences of impact in SMC. In the manuscript, we have now included the references to each parameter values in a table (as per your suggestion, see the answer below) and added the following justification in the discussion:

***There are also fixed model parameters, estimated as part of previous OpenMalaria calibration work: (e.g. species-specific efficacy of nets [36], efficacy of treatments [44, 51], and immunity development mechanisms [29, 55]), but these refer mainly to the biology of malaria and are unlikely to differ in Benin.*

 As it is a pure modelling study, the paper could be made more general by trying to illustrate what is the generality that targeting 0-5 is better than adding 5-10. Identifying what conditions make it so.**
Thank you for this important point. In our analysis, the results are very likely driven by the high transmission intensity in all the Benin departments considered for the extension scenarios. Under such endemicity levels, severe cases and deaths, which are the main target for SMC according to WHO, occur primarily in younger children. Therefore, targeting 0-5 rather than 5-10 was identified as more beneficial. However, we prefer to refrain ourselves from extrapolating our conclusions to any geography, as unpublished analyses comparing SMC extensions in different countries are conducted in our group, and they found that targeting more children under 5 is not necessarily better than including those aged 5-10, depending on the geographies. Such general conclusions are therefore beyond the scope of this manuscript and will be the object of future work and publications.

As mentioned above, we have included these considerations in the revised manuscript. We have highlighted in the introduction why both extensions could be beneficial, and that context is critical to determine which extension to choose. We have also expanded the discussion section to better highlight the epidemiological factors that explain our results.

**other points :

 ABSTRACT
 'likely more cost-effective' should be removed. You did not perform cost effectiveness analysis.**
Thank you for this remark. We have now removed all mentions of cost-effectiveness in the manuscript. In the abstract, we now write:

*In Benin the geographic extension would be more impactful than the demographic extension* ***both in absolute number of severe cases averted and per child protected***

We wanted to stress that modelling allows not only to identify the best option, but also to quantify by how much it outperforms the other.

**Are these the same children receiving SMC during the season or is it sampled on each of the 4 rounds? does it change?**
Thank you for pointing out this need for clarification. At each round 80% of the children are randomly sampled to receive SMC, which means all children are equally likely to receive the intervention at each round. This probably does not reflect exactly the reality, where some children are much more likely to receive SMC than others, but we did not have data on how many children had received which number of doses. Also, other studies had shown that for other chemoprevention like mass drug administration (MDA), there was very similar impact between allocating to the same children or randomly each time (Brady 2017). As a result we assumed our assumption was still relevant.

We clarified it in the Methods section:

*To match* ***the lowest reported coverage*** *from the 2021 SMC campaign, 80% of eligible children under 5 were randomly selected to receive SMC at each of the four rounds from July to October.* ***This means each child was equally likely to receive SMC at each round.***

and added a section in the Discussion to explain why this random selection is a conservative assumption

***At each round 80% of eligible children were randomly selected to receive SMC, so each child was equally likely to receive the intervention at each round, when in practice some children are systematically missed. In Baba et al. [7], the percentage of children who received four treatments of SMC was higher than the mean monthly coverage to the power of four in all countries but Chad, indicating that the selection of children is not random [25]. However we did not have data on the proportion of children who received each number of rounds, and by randomly selecting 80% of children at each round we obtain on average 41% of children receiving all four rounds, which is a conservative assumption when comparing to the 51.8% children who received all four doses according to the 2021 SMC campaign report. A modelling study [21] found that for another chemoprevention method (Mass Drug Administration) there was little difference in impact between allocating the intervention to the same children or randomly at each round.***

**It is difficult to follow what is necessary to establish a scenario (nets, treatment, efficacy).
 A table listing parameters and sources would be welcome.**
 Thank you for this very valuable suggestion! We have added the following table at the beginning of the Methods section:

***Table 1: Model parameters and data sources***

| ***Parameter*** | ***Value*** | ***Source*** |
| --- | --- | --- |
| ***Vector species contribution*** | **Anopheles gambiae sensu lato *90%***  **Anopheles funestus *10%*** | ***[32]*** |
| ***Proportion of bites occurring outdoors*** | ***20% for* Anopheles gambiae sensu lato**  ***10% for* Anopheles funestus** | ***Computed from human sleeping patterns and mosquito rhythms from Benin and neighbouring countries using the AnophelesModel R package [33]*** |
| ***Seasonality of transmission*** | ***Based on monthly incidence by department, averaged between 2011 and 2017 with a 15-day lag*** | ***Cases from national surveillance [34], population from census [35]*** |
| ***Types of nets*** | ***Only pyrethroid-only nets until 2020***  ***Mixture of pyrethroid-only nets and PBO in 2020, and of pyrethroid-only nets, PBO and chlorfenapyr nets in 2023*** | ***Matching*** |
| ***Efficacy of nets*** | ***From experimental hut trials From 2016, pyrethroid-only nets are modelled as a physical barrier only, to account for widespread insecticide resistance*** | ***[36] PBO and chlorfenapyr nets are both modelled as restoring susceptibility to insecticide*** |
| ***Net use*** | ***Specific values per year and per department.***  ***National average value: 6.5% in 2000, 71.1% in 2017*** | ***2000 – 2010: MAP [37]  2011 – 2023: based on latest household survey at distribution time [38, 39]*** |
| ***Net durability*** | ***Before 2020: 3-year half-life After 2020: Depending on fabric: 2-year halflife for polyester nets (pyrethroid-only nets, Interceptor G2 and PermaNet 3.0), 1.6 for polyethylene (DuraNet)*** | ***Before 2020 [40] After 2020 [41]*** |
| ***IRS implementation*** | ***IRS active ingredient, population coverage and month of spraying estimated from PMI Malaria Operational Plans***  ***Coverages between communes and years ranged from 47% to 90%*** | ***[42]*** |
| ***IRS efficacy*** | ***From experimental hut trials*** | ***[33, 43]*** |
| ***Effective treatment coverage*** | ***Uncomplicated cases: specific values per year and per department***  ***National average value: 22.8% in 2001, 28.1% in 2018***  ***Severe cases: 48%*** | ***Uncomplicated cases: methodology by [44], with data from [38, 39] Severe cases [45]*** |
| ***SMC efficacy*** | ***25 days blood clearance*** | ***Calibrated to match effect size in [46]*** |
| ***SMC coverage*** | ***80% of children at random at each of the four rounds from July to October*** | ***Reported coverage from the 2021 campaign*** |
| ***Age structure*** | ***Benin census*** | ***[35]*** |
| ***Importation rate*** | ***10 infections per 1000 people per year in each commune*** | ***[47]*** |
| ***Parasite detection limit*** | ***100 parasites per microliter*** | ***[48]*** |
| ***Malaria prevalence*** | ***Geospatial estimates of prevalence in children aged 2 to 10, based on surveys in children under 5***  ***National prevalence in children under 5 in Benin in 2017: 36.4%***  ***Alibori: 36%***  ***Atacora: 53.9%***  ***Borgou: 51.3%***  ***Collines: 41.6%***  ***Donga: 40.2%*** | ***[38, 49]*** |

**No data is presented in the main text to illustrate that the model captures the underlying situation satisfactorily.
 Fig A.9-A.13 does not show a particular good fit to the data: the average is indeed calibrated, but the model does not seem to reproduce the observed trends. How did the authors decide that the model provided a satisfactory fit to the data? Would the lack of fit in trends alter the conclusions ?**

Thank you for this important point. We have now included a full section in the Results to describe how our model reproduces malaria trends in Benin and have provided more justifications about the quality of the fit.

At the national level, our model fits the beginning and the end of the time period but the rebound in malaria prevalence is shifted to later years in our model compared to MAP’s prevalence estimates, leading to a difference in trends between 2012 and 2017. MAP’s prevalence estimates are themselves the result of a global geospatial model with its own uncertainties and not a direct measurement of malaria prevalence trends in Benin. The only measurements available are three cross-sectional population surveys, representative at the department level (against which we validate in Figure 3B).

In our mechanistic model, we can explain the factors responsible for the predicted trend. The large decrease in malaria transmission after 2010 is largely driven by the scale-up of control interventions, in particular ITNs. After 2016, the re-increase is due to 1/ a reduction in ITN efficacy due to insecticide resistance that we introduce in 2016 (when resistance was first reported in the country) and 2/ from the reduced acquired immunity in populations protected by ITNs over long periods of time, documented in previous modelling analyses with OpenMalaria (Briet and Penny 2013).

Our approach is fully mechanistic: we explicitly account for the factors which could influence malaria epidemiology in Benin by relying on country data for timing of interventions and appearance of insecticide resistance. Given the limited available data on prevalence trends and the complexity of the OpenMalaria individual-based model, we adopted a simple calibration methodology, fitting only one parameter to avoid overfitting but making good efforts to propagate uncertainty in the input data. We believe that this approach, even if it leads to imperfect fitting, is more transparent in that all prevalence changes can be mechanistically explained by model assumptions.

In the end what drives the conclusion towards the geographic extension seems to be the large proportion of severe cases occurring in young children, which is due to the estimate high transmission intensity. Even if our EIR calibration is subject to uncertainty, we know from the very high prevalence in northern Benin (36 to 54% in children under 5 according to the DHS 2017) that it is indeed high. Therefore, even if we don’t perfectly reproduce the past trends between 2012 and 2017, we are confident that the main conclusion of the manuscript (superiority of the geographic extension) is not affected by this limitation.

The quality of the calibration may have influenced the prioritisation analysis more, if some zones had been incorrectly estimated to have very high or very low transmission intensities compared to others. This is why we made efforts to propagate model and calibration uncertainties (see answer below for further methodological details). In the end, the uncertainty level in the prioritisation analysis prevented us from drawing any prescriptive recommendation on that aspect, as we transparently reported.

In the manuscript, the new section within the Results is as follows:

***In each of Benin’s 77 communes, we calibrated the OpenMalaria model to reproduce the trend in annual malaria prevalence between 2006 and 2019, as estimated by the Malaria Atlas project [49]: the resulting modelled prevalence at the national level is displayed in Figure 3A (commune-level results are presented in Appendix A). The model captures the overall prevalence trend, which decreased from 44% (41 – 46) in 2006 to 31% (28 – 34) in 2020, although a rebound in the years 2010 was observed (Figure 3A). This rebound happens later in our model predictions compared to MAP’s estimates, leading to a difference in trends between 2012 and 2017, but overall, uncertainty intervals overlap for all years except 2015 and 2016. MAP’s yearly prevalence estimates are based on DHS and MIS surveys which are conducted during a few months and are powered to be representative at the department level [38, 39]. When comparing modelled prevalence in each department during the same months to those surveys, we obtained an adjusted R^2^ of 0.59 (Figure 3B). However the 2015 MIS survey [39] was not used to produce MAP’s prevalence estimates, and when comparing the modelled prevalence against the survey prevalence for the 2011 and 2017 surveys only, we found a strong correlation (R^2^ = 0.79).***

**Figure 3: (A) Estimated (green, Malaria Atlas Project [49]) and modelled (grey) yearly malaria prevalence in children under 5 aggregated at the national level. (B) Modelled malaria prevalence in children under 5 against DHS and MIS surveys in each department [38, 39], matched by survey period. Ribbon (A) and error bars (B) represent uncertainty on intensity of transmission as well as model stochasticity, as described in the Methods section.**

**Predictions of the trends in malaria cases and incidence between 2000 and 2023 are displayed in Figure 4. The total number of predicted malaria episodes increased from 8.1 (8 – 8.3) million in 2000 to 17.8 (17.5 – 18) million in 2023. This trend is largely driven by population growth, as the population in Benin more than doubled from 6.4 million inhabitants in 2000 to 14.1 million in 2023 [35]. Modelled malaria incidence in children under 5, which is not affected by population growth, decreased from 3392 (3269 – 3466) malaria episodes per 1000 children in 2000, to 2378 (2196 – 2492) in 2023, with a strong decrease after 2010 to reach its lowest value in 2016 and a re-increase since. A similar trend, although more moderate, was observed in all-age incidence. The strong incidence reductions after 2010 can be attributed in the model to the large scale deployment of malaria interventions, especially ITNs. The re-increase is due to the reduction in ITN efficacy due to insecticide resistance in 2016 and from the reduced acquired immunity in populations protected by ITNs over long periods of time [52].**

**Since the model was calibrated to prevalence, we could validate predictions of malaria cases to other sources, such as the World Malaria Report (WMR) 2024 [1] and routine surveillance data [34]. As expected, the number of modelled malaria episodes was substantially higher than both the WMR and routine data, due to OpenMalaria’s large threshold for clinical case definition and the double counting of illnesses lasting over 30 days [51]. Treated cases, on the other hand, are infections which were detected and treated by the health system according to the effective treatment assumptions. For 2023, the model predicted 4.5 (4.4 – 4.7) million treated cases, whereas the World Malaria Report estimated 5.1 (3.2 – 7.7) million cases [1]. Modelled treated cases account for the inefficiencies in the case management cascade, but nationally reported cases are even lower since systematic testing and reporting of cases is still being improved in Benin.**

**Figure 4: Trends in absolute numbers (A) and incidence (B) of modelled indicators. (A) Absolute numbers of modelled malaria episodes and treated cases in all ages, as well as cases reported by Benin and estimates from the World Malaria Report (WMR) 2024 [1]. (B) Incidence of modelled malaria episodes per 1000 persons at risk in all age groups and children under 5. Ribbons represent uncertainty on intensity of transmission as well as model stochasticity, as described in the Methods section.**

Additionally, we added more justifications in the limitation paragraph of the discussion:

*EIR was estimated based on prevalence data, and we assumed that all changes in malaria burden were due to interventions*, ***which made the model less flexible to reproduce all past trends perfectly***. *In practice, climate or socio-economic changes also play a role****, but they should influence similarly all considered scenarios and hence not alter our conclusions which rely only on relative comparisons****.*

**In the priorisation study, what could be the differences leading to a change in impact? Aren't those territories modelled all the same, using the same model ?**
These departments are modelled as having different characteristics (seasonality) and different histories of interventions (net use and treatment coverage). There were also differences at the commune level (second administrative level) in the types and thus efficacy of nets distributed in 2020 and 2023 (see Figure A.5 in Appendix), as well as IRS in Donga (Figure A.6). Since the calibration was done at the commune level to prevalence data by commune, we also obtained different transmission intensities: point estimates of EIR ranged from 38 to 162 infective bites per person per year.

We were thus expecting to see some differences in the impact of SMC – but found that all these differences in the modelling of the different zones were still not resulting in a visible difference of impact, given the level of model and data uncertainty. However we thought it was important to stress that modelling does not necessarily give a clear answer to each question, and that it should not be the only element taken into account by the decision-makers. Rather, cost or availability of health workers could guide this decision. We expended the corresponding section in the discussion to better explain why we expected a difference:

***The health zones within the geographic extension were modelled with different seasonalities, history of interventions and transmission risk, but episodes and*** *severe cases averted per targeted child were similar* ***given the level of uncertainty – meaning the differences we took into account were not strong enough to drive a differentiated impact given the level of model and data uncertainties.*** *Other considerations, such as the cost of extending to each zone, could be taken into account to prioritise where to implement SMC first. In the end, the availability of community health workers guided the order of implementation.*

**Reviewer #2: This work presents a study estimating the impact of interventions planned to control malaria transmission in Benin using a mathematical modeling approach. The problem is undoubtedly relevant, and the approach is valid, despite certain limitations acknowledged by the authors. However, in my view, the study does not adequately describe the methodology.**

Thank you for these encouraging comments. As mentioned above, we have substantially expanded the Methods section. In particular, we have provided additional details on model parameters, calibration methodology and results, scenarios definition and scenario comparison as explained below.

**Given a baseline, which is calibrated in the model, the authors could have provided the baseline numbers. I understand that the effect of planned interventions is estimated using parameters such as ITN coverage and drug treatment. However, it is unclear whether the planned interventions are already in place or still in development. My primary concern revolves around the concept of a baseline. The study compares the impact of additional layers of interventions with the planned interventions.**

Thank you for pointing this need for clarification. The model represents malaria transmission in Benin both in the past (2000-2023) and in the future (2024-2026).

For the past, the model includes all interventions that were deployed, and we parameterised deployment dates, coverages, and intervention characteristics as they happened from the available data.

For the period 2024-2026, a number of interventions were already planned to happen independently of the choice of the SMC extension strategy, namely ITN distributions, PMC and SMC among children under 5 in the previously targeted departments. These interventions were not yet deployed when the modelling work was conducted in 2023/2024, but their implementation was already confirmed. It was important to include them when comparing future scenarios of SMC extension, as they could influence the relative impact of SMC in different areas.

In order to improve clarity, we have revised the manuscript as follows.
First, we have added further details to describe the malaria situation prior to the future intervention scenarios, in a new section describing our calibration results (see answers below). For the year 2023, the text now mentions:

***For 2023, the model predicted 4.5 (4.4 – 4.7) million treated cases, whereas the World Malaria Report estimated 5.1 (3.2 – 7.7) million cases [1].***

Second, in order to better describe the comparator scenario in the future simulations, we have revised Figure 2 as follows:

**Figure 2: Future scenarios for the period 2024 to 2026. All communes receive additionally next-generation nets (Interceptor G2) in 2026 in all three scenarios.**

And added the following sentence in the text:

***Some interventions were already scheduled by the NMCP, such as continuing SMC campaigns for*** *children under 5 in Alibori and Atacora* ***and distributing next-generation*** *nets in 2026* ***in the whole country. The*** *Plus Project, a pilot study of perennial malaria chemoprevention (PMC)* ***targeting children until 2 years, had also started in some communes (Figure 2). These interventions, although not yet fully deployed when the modelling work was conducted, were confirmed to happen and needed to be included in the model for a comprehensive representation of malaria control over the period 2024-2026.***

**Another issue is that the comparison involves uncertainties on both sides—the effect of the planned interventions and the effect of the additional interventions.
 If this interpretation is correct, the methodology section should provide a clearer explanation of how these comparisons are made.**

Thank you for this comment. In order to calculate cases averted, we matched simulations by seed and simulated EIR and computed the difference in number of cases between the baseline scenario (planned interventions only) and each extension scenario. To obtain the mean number of cases averted, we averaged the cases averted across seeds for the EIR point estimate. For the credible intervals, we compute the minimum (and maximum) cases averted across all seeds and EIR estimates. We have now explained the comparison in much more details and with mathematical formulas in the Methods section:

***We computed epidemiological indicators*** $\boldsymbol{I}$ ***(episodes, severe cases and deaths)*** *averted by each extension scenario by comparing those occurring with only the planned interventions* ***to those with either the demographic or the geographic extension of SMC. Each simulation is uniquely defined by a commune, a stochastic replicate*** $\boldsymbol{s\in\{1,\ldots,10\}}$***, an EIR value*** $\boldsymbol{E\in\{}\boldsymbol{E}^{\boldsymbol{low}}\boldsymbol{,}\boldsymbol{E}^{\boldsymbol{point}}\boldsymbol{,}\boldsymbol{E}^{\boldsymbol{up}}\boldsymbol{\}}$ ***and a scenario. We computed the mean averted indicator*** $\boldsymbol{A}_{\boldsymbol{extension}}^{\boldsymbol{point}}$ ***between the planned interventions and an extension scenario as such:***

$$\boldsymbol{A}_{\boldsymbol{extension}}^{\boldsymbol{point}}\boldsymbol{=}\underset{\boldsymbol{s\in\{1,\ldots,10\}}}{\boldsymbol{mean}} \boldsymbol{(}\boldsymbol{I}_{\boldsymbol{planned}}^{\boldsymbol{s,}\boldsymbol{E}^{\boldsymbol{point}}}\boldsymbol{-}\boldsymbol{I}_{\boldsymbol{extension}}^{\boldsymbol{s,}\boldsymbol{E}^{\boldsymbol{point}}}\boldsymbol{)}$$

***and the lower bound of the credible interval***

$$\boldsymbol{A}_{\boldsymbol{extension}}^{\boldsymbol{low}}\boldsymbol{=}\min_{\begin{aligned} \boldsymbol{s\in}\left\{ \boldsymbol{1,\ldots,10} \right\} \\ \boldsymbol{E\in\{}\boldsymbol{E}^{\boldsymbol{low}}\boldsymbol{,}\boldsymbol{E}^{\boldsymbol{point}}\boldsymbol{,}\boldsymbol{E}^{\boldsymbol{up}}\boldsymbol{\}} \end{aligned}} \boldsymbol{(}\boldsymbol{I}_{\boldsymbol{planned}}^{\boldsymbol{s,E}}\boldsymbol{-}\boldsymbol{I}_{\boldsymbol{extension}}^{\boldsymbol{s,E}}\boldsymbol{)}$$

***(respectively taking the maximum for the upper bound). We computed the extremum over the EIR as well as the stochastic replicates since for close EIR values, a lower EIR can produce a slightly higher indicator.***

***We summed each averted indicator between 2024 and 2026 over all communes of Alibori, Atacora, Borgou (except Bembereke-Sinende), Collines and Donga.***

**Furthermore, in the results, the overall epidemiological profile and baseline should be presented, leaving a reader unfamiliar with malaria in Benin without a clear understanding of the number of cases involved.**

Thank you for this comment. As mentioned above, we have added a full section in the results to describe malaria trends in Benin since 2000. It includes a figure (Figure 4 in the revised manuscript) showing the trend in cases and incidence in Benin, as well as the numbers of cases as mentioned above.

**Also, an analysis of cost effectiveness was not done, instead the impact was estimated via numbers of avoided cases. I suggest refraining from taking results on the basis of cost-effectiveness.**

Thank you for pointing this out, we have removed all mentions of cost-effectiveness in the manuscript. The results sentence now reads

*Since SMC is recommended to “children belonging to age groups at high risk of severe malaria” [4], the* ***geographic*** *extension* ***appears far preferable as it averts 9 times more severe cases in absolute numbers and 4.5 times more severe cases per targeted children.***

**Additional comments:

 Introduction

 The statement about the goal says that the goal is to verify which extensions on interventions are cost-effective, however a rigorous cost-effectiveness analysis was not performed.**

Thank you for pointing this out, the sentence now reads

*Beyond determining which of the two extension strategies would save more lives, understanding their relative impact would enable the NMCP to weigh this likely impact* ***against the required implementation effort.***

and the goal sentence:

*Our goal was to determine which extension of SMC would have more impact in Benin,* ***to quantify this difference in impact*

 Methodology

 The methodology should include a description of the study area—in this case, the Benin population—and how cases have been reported over the past years (including sources of data).**

Thank you for this comment. As mentioned above, we added a section in the Results to describe the trend in malaria cases and incidence in Benin since the year 2000, comparing our model predictions to several data sources, including the World malaria report and the routine surveillance data:

**Predictions of the trends in malaria cases and incidence between 2000 and 2023 are displayed in Figure 4. The total number of predicted malaria episodes increased from 8.1 (8 – 8.3) million in 2000 to 17.8 (17.5 – 18) million in 2023. This trend is largely driven by population growth, as the population in Benin more than doubled from 6.4 million inhabitants in 2000 to 14.1 million in 2023 [35]. Modelled malaria incidence in children under 5, which is not affected by population growth, decreased from 3392 (3269 – 3466) malaria episodes per 1000 children in 2000, to 2378 (2196 – 2492) in 2023, with a strong decrease after 2010 to reach its lowest value in 2016 and a re-increase since. A similar trend, although more moderate, was observed in all-age incidence. The strong incidence reductions after 2010 can be attributed in the model to the large scale deployment of malaria interventions, especially ITNs. The re-increase is due to the reduction in ITN efficacy due to insecticide resistance in 2016 and from the reduced acquired immunity in populations protected by ITNs over long periods of time [52].**

**Since the model was calibrated to prevalence, we could validate predictions of malaria cases to other sources, such as the World Malaria Report (WMR) 2024 [1] and routine surveillance data [34]. As expected, the number of modelled malaria episodes was substantially higher than both the WMR and routine data, due to OpenMalaria’s large threshold for clinical case definition and the double counting of illnesses lasting over 30 days [51]. Treated cases, on the other hand, are infections which were detected and treated by the health system according to the effective treatment assumptions. For 2023, the model predicted 4.5 (4.4 – 4.7) million treated cases, whereas the World Malaria Report estimated 5.1 (3.2 – 7.7) million cases [1]. Modelled treated cases account for the inefficiencies in the case management cascade, but nationally reported cases are even lower since systematic testing and reporting of cases is still being improved in Benin.**

**Figure 4: Trends in absolute numbers (A) and incidence (B) of modelled indicators. (A) Absolute numbers of modelled malaria episodes and treated cases in all ages, as well as cases reported by Benin and estimates from the World Malaria Report (WMR) 2024 [1]. (B) Incidence of modelled malaria episodes per 1000 persons at risk in all age groups and children under 5. Ribbons represent uncertainty on intensity of transmission as well as model stochasticity, as described in the Methods section.**

**How was the EIR in 2000 obtained? It is unclear whether it comes from a previous publication or was estimated through a specific methodology. The authors mention that lower and upper bound estimates for EIR were used to account for uncertainty, but how were these estimates determined?**
Thank you for this comment. We use a pseudo-likelihood method to estimate the mean EIR which allows us to best reproduce the prevalence estimates in each commune, and we have adapted a profile likelihood approach from Ionides et al. to estimates the upper and lower bound EIR. We have brought the description of the methodology to the main text of the article, after the description of the interventions.

*The transmission level, represented by the pre-intervention Entomological Inoculation Rate (EIR) in 2000, was adjusted for each commune to match the time series of past prevalence estimates* ***for years 2006 to 2019 provided by the Malaria Atlas Project (MAP) [49].***

***For each department, we computed a grid of simulations sharing the same history of interventions and geographical characteristics but with EIR values ranging from 1 to 250 (with a step of 1 until 10, and a step of 2 afterward), and 10 stochastic replicates. The associated OpenMalaria simulations for prevalence over the years 2006 to 2019 are noted*** $\boldsymbol{X}^{\boldsymbol{sim, \theta}}\boldsymbol{=}\left( \boldsymbol{X}_{\boldsymbol{i}}^{\boldsymbol{sim,\theta}} \right)_{\boldsymbol{i\in\{2006,\ldots,2019\}}}$***, with*** $\boldsymbol{\theta}$ ***denoting each simulation’s unique set of parameters (including the initial EIR).***

***MAP’s prevalence estimates [49] are noted*** $\boldsymbol{X}^{\boldsymbol{obs}}\boldsymbol{=}\left( \boldsymbol{X}_{\boldsymbol{i}}^{\boldsymbol{obs}} \right)_{\boldsymbol{i\in\{2006,\ldots,2019\}}}$ ***and their associated 95%-confidence intervals*** $\boldsymbol{(}\boldsymbol{X}_{\boldsymbol{i}}^{\boldsymbol{low}}\boldsymbol{,}\boldsymbol{X}_{\boldsymbol{i}}^{\boldsymbol{up}}\boldsymbol{)}$ ***were used to compute an estimated standard deviation*** $\boldsymbol{\sigma=}\left( \boldsymbol{\sigma}_{\boldsymbol{i}} \right)_{\boldsymbol{i\in\{2006,\ldots,2019\}}}$ ***as*** $\boldsymbol{\sigma}_{\boldsymbol{i}}\boldsymbol{=}\frac{\boldsymbol{max(}\boldsymbol{X}_{\boldsymbol{i}}^{\boldsymbol{obs}}\boldsymbol{-}\boldsymbol{X}_{\boldsymbol{i}}^{\boldsymbol{low}}\boldsymbol{,}\boldsymbol{X}_{\boldsymbol{i}}^{\boldsymbol{obs}}\boldsymbol{+}\boldsymbol{X}_{\boldsymbol{i}}^{\boldsymbol{up}}\boldsymbol{)}}{\boldsymbol{1.96}}$***. For each simulation in the grid, a pseudo-likelihood comparing model prevalence to MAP prevalence is computed as***

$$\boldsymbol{L}\left( \boldsymbol{X}^{\boldsymbol{obs}}\boldsymbol{,}\boldsymbol{X}^{\boldsymbol{sim, \theta}} \right)\boldsymbol{=}\boldsymbol{\Pi}_{\boldsymbol{i=2006}}^{\boldsymbol{2019}}\boldsymbol{\Phi}_{\boldsymbol{X}_{\boldsymbol{i}}^{\boldsymbol{sim, \theta}}\boldsymbol{,}\boldsymbol{\sigma}_{\boldsymbol{i}}}\left( \boldsymbol{X}_{\boldsymbol{i}}^{\boldsymbol{obs}} \right)$$

***where*** $\boldsymbol{\Phi}_{\boldsymbol{\mu,,\sigma}}$ ***represents the probability density function of a normal distribution with mean*** $\boldsymbol{\mu}$ ***and standard deviation*** $\boldsymbol{\sigma}$***. Because the standard deviation associated with each observation point is accounted for, the pseudo-likelihood assigns more importance to observations with lower uncertainty. The EIR value with highest log-likelihood (***$\boldsymbol{E}^{\boldsymbol{point}}$***) is selected as point estimate.***

***An uncertainty estimate is computed using the profile likelihood approach by Ionides et al. [50] adapted to this particular use case. In Ionides et al. [50], a local quadratic approximation is fitted on the smoothed pseudo-likelihood and used to calculate the threshold used in the definition of the confidence intervals. For each commune, the local window for the quadratic approximation was defined by the EIR values for which*** $\frac{\left| \boldsymbol{E}^{\boldsymbol{point}}\boldsymbol{-EIR} \right|}{\boldsymbol{E}^{\boldsymbol{point}}}\boldsymbol{\leq}\boldsymbol{0.5}$ ***and the quality of the quadratic approximation was assessed graphically. Thanks to this method, we obtained upper and lower uncertainty bounds on the initial EIR (***$\boldsymbol{E}^{\boldsymbol{low}}$ ***and*** $\boldsymbol{E}^{\boldsymbol{up}}$***) for each commune.***

***In the future scenarios, for each of Benin’s 77 communes, OpenMalaria was run for*** $\boldsymbol{E}^{\boldsymbol{point}}\boldsymbol{,}\boldsymbol{E}^{\boldsymbol{low}}$ ***and***$\boldsymbol{E}^{\boldsymbol{up}}$***, in order to propagate the uncertainty in transmission intensity due to prevalence data fitting. Each future simulation also includes 10 replicates to account for model stochasticity. The final uncertainty intervals represent the ensemble of both sources of uncertainty.***

**Why were 10 infections per 1,000 people considered for imported cases?**
Thank you for pointing out that a justification was missing. Previous modelling work has relied on a study on importation rates from mainland Tanzania to Zanzibar (Tatem et al.) which estimated the importation rate ranged from 1 to 12 imported infections per 1000 people per year. With 10, we are on the conservative side. Overall, this parameter has a very limited influence in a highly endemic setting like northern Benin, where malaria transmission is largely locally sustained. We have added the citation in the main text:

*We also assumed 10 infections per 1000 people per year were imported in each commune****, a a conservative assumption [47].***

**What is the evaluation period? Are all simulation scenarios based on a two-year period? This was not clear. I suggest providing a table or a descriptive list of the scenarios.**
We have added a sentence at the beginning of the “Simulation of SMC extensions” section to explicit the evaluation period :

***The future scenarios were simulated from 2024 to 2026, which was the period of interest defined by the NMCP as the funding period.***

Additionally, as you suggested below, we have redrawn Table 1 as a figure (Figure 2) with the explicit scenario names, to show clearly which scenarios contains which interventions.

***Figure 2: Future scenarios for the period 2024 to 2026. All communes receive additionally next-generation nets (Interceptor G2) in 2026 in all three scenarios.***

**Table 1 contains figures—perhaps it would be better as a panel of figures.**
Thanks for the suggestion: we have replaced the table by a panel of figures.

**The number of newly targeted children is used a proxy for cost-effectiveness. A truly cost-effectiveness analysis would involve the financial costs. I understand that the study quantifies the impact and the number of newly targeted children is a measure of the effort of the additional interventions.**

We have removed the term “cost-effectiveness” from the entire manuscript. Thank you for this phrase which we have reused in the section:

*The number of newly targeted children is also* ***used as a measure of the effort of extending SMC.***

**Results

 I believe the baseline is missing from the manuscript. Possibly a figure showing the time series of cases up to 2023, or a table with demographic information (such as areas, cases, and severe cases) should be included.**

Thank you for this suggestion. As mentioned above, Figure 4 in the revised manuscript now shows the time series of both modelled and reported cases and the text of the results section comments the trends and its drivers.

**Additionally, a profile of the population exposed to malaria could be helpful, possibly presented in a table. The supporting info material has additional information that could bring this notion.**
Thanks for pointing us to this need for contextual information. As mentioned above, we substantially expanded the results section to include a graphic of the trend in malaria cases since 2006, with its interpretation. In the Methods section, we have also now a full table that summarises the main parameters of the model, and now includes summary data on environmental factors and intervention coverages. It also includes the national and departmental prevalence as measured in the 2017 Demographic and Health Survey (results from the 2022 survey are not available yet) to present the higher burden in the north of the country.

**For Figure 2 (and subsequent figures), what is the time interval?**
The time interval is 2024-2026, corresponding to the funding period. We have now consistently specified it in the main legend of each figure.

**Figure 4 introduces terms that were not previously defined, such as NKP and DAGLA. The caption, “Error bars represent uncertainty in transmission intensity as well as model stochasticity,” suggests that uncertainty arises from both factors, possibly implying they are independent. However, I would argue that stochasticity is used in the model to describe this uncertainty. Therefore, I suggest rephrasing this to avoid misleading interpretations.**
Thank you for pointing this out, we have edited the legend:

***Each acronym stands for one health zone (made of one or several communes): NKP: Nikki-Kalale-Perere; PN: Parakou-N'Dali; TCH: Tchaourou; DAGLA: Dassa-Zoume-Glazoue; SABA: Savalou-Bante; SAO: Save-Ouesse; BLA: Bassila; DCO: Djougou-Copargo-Ouake***

Our uncertainty takes into account both the uncertainty in transmission intensity (by propagating the uncertainty in the prevalence estimates we are fitting to) and the model stochasticity. These factors are incorporated into the uncertainty bounds on the EIR. As mentioned above, we have brought the description of the calibration methodology to the main text of the article.

**Discussion

 In the paragraph on limitations in the Discussion, the authors should explain why these limitations can be accepted—whether the biases are expected to be minimal or if a more conservative approach is warranted.**

Thank you for this remark. We have edited the paragraph as follows:

*This work has* ***nonetheless some*** *limitations. It relies on a model of malaria transmission, which is a simplification of reality. Many parameters of the model were tailored to match local data, which implies that collected data was trusted to reflect reality. EIR was estimated* ***based on*** *prevalence data, and we assumed that all changes in malaria burden were due to interventions,* ***which made the model less flexible to reproduce all past trends perfectly****. In practice, climate or socio-economic changes also play a role,* ***but they should influence similarly all considered scenarios and hence not alter our conclusions which rely only on relative comparisons.*** *Given the lack of granularity in the data, some* ***conservative*** *uniformity assumptions were* ***also*** *made: net usage and treatment seeking were assumed uniform across each department,* Anopheles *species composition, and apparition of resistance identical across the entire country.* ***Even though geographical variations in these factors could be relevant, the absence of reliable data prevents us from including such variations in our model: our conclusions thus provide a recommendation on the best strategy given the available evidence, as required for decision-making****. There are also fixed model parameters****, estimated as part of previous OpenMalaria calibration work: (e.g. species-specific*** *efficacy of nets* ***[36], efficacy of*** *treatments* ***[44, 51], and*** *immunity development mechanisms* ***[29, 55]), but these refer mainly to the biology of malaria and are unlikely to differ in Benin. The variables with the strongest influence on the local impact of SMC, namely seasonality, other interventions deployed at the same time as SMC, and SMC coverage, were all estimated from Benin data.***

**I believe the issue of drug resistance should be addressed. This is important when expanding interventions as proposed here.**

We agree that drug resistance needs to be continuously monitored to preserve the efficacy of SMC. Svigel et al. (2021) found that resistant alleles to sulfadoxine-pyrimethamine were not widespread in Benin and supported the continued use of SP for chemoprevention. Moreover, a recently published modelling study (Masserey 2024) showed that the spread of quintuple mutants was slowest when administering four rounds of SMC to children under 5 years of age, which is the strategy we recommended and that is being implemented in Benin. Provided that regular monitoring is in place, we believe the risk of resistance emerging is controlled.

We have added the following paragraph to the discussion:

***The model does not account for the possible emergence of resistance to sulfadoxine-pyrimethamine, but a study using samples collected in 2017 in Benin [56] did not find evidence of widespread resistance in the country. Moreover, modelling evidence [57] indicates that SMC leads to a slow spread of resistance, especially when SMC is administered during four rounds and to children under 5, which happened to be the recommended strategy. The risk of SP resistance developing thus seems minor over the considered time frame.***
