# Supplementary material for: Supporting evidence-based decisions about the geographic and demographic extensions of seasonal malaria chemoprevention in Benin: A modelling study
Source: PLOS Glob Public Health. 2025 May 19;5(5):e0004509. doi: 10.1371/journal.pgph.0004509 (PMC12088001; doi:10.1371/journal.pgph.0004509)
Supplement: Attachment — Submitted filename: Response to Reviewers_2nd revisions.docx [file pgph.0004509.s004.docx]

March 17th, 2025

Dear Editor,

We thank the reviewers for their second read of our work which highlighted remaining unclear points. We have edited the article to address all the comments and believe the readability and understanding has improved thanks to them. We provided a detailed answer to comments below, as well as edits in the text in bold.

We thank you very much for your time and consideration.

Sincerely,

Emilie Pothin, on behalf of all authors

**Reviewer #2: The manuscript improved significantly with more information on the methodology, updates on the results, and several other aspects.**

**I believe now it lacks conveying in the abstract the notion that the geographical extension is advantageous because of the effect on children ages 0-5.**

**I also recommend a revision on some points that appear in the Results, however they suit better in the Discussion.**

**Other comments below:**

**- The abstract has to reflect the point that due to the frequency of severe cases in ages 0-5, the geographical extension had better performance.**

Thank you for this important point. We have modified both the Findings and Conclusions parts of the abstract to stress this point:

*The model predicted that between 2024 and 2026 the geographic extension of SMC would avert at least four times more severe malaria cases and five times more direct malaria deaths per targeted child than the demographic extension.* ***Indeed, most severe cases are concentrated in children under 5 in all departments of interest, as malaria burden remains high in this region.***

***Due to the high malaria burden in northern and central Benin****, the geographic extension would be more impactful than the demographic extension both in absolute number of severe cases averted and per child protected*

**- Line 47: provide the location for the number of cases**

We added the term “worldwide” in the sentence (line 48 in the revised manuscript).

**- Line 171: remove “by far”**

Thank you, this has been addressed line 132 in the revised manuscript.

**- Line 204: correct “ Once an individual seeks treatment, they do not necessarily”**

Thank you, this has been addressed line 160:

*“Once* ***individuals seek*** *treatment, they do not necessarily”*

**- Be more clear in the sentence: “Because the standard deviation associated with each observation point is accounted for, the pseudo-likelihood assigns more importance to observations with lower uncertainty.” I think it requires a better understanding both in terms of “standard deviation associated with an observation point” and “more importance to lower uncertainty”.**

We decided to remove this sentence, as it was only a comment on the previous formulae, and the formulae themselves provide a comprehensive and sufficient description of the methodology.

**- It is unclear why simulations were initially run with 50,000 of population size if later the actual population sizes were used, which makes sense.**

This was a trade-off between a larger population to measure rare events such as deaths and severe episodes, while keeping a reasonable runtime (each of the 4000 simulations requiring almost two hours of computation). Additionally, we wanted to avoid simulating a larger population size than the real population of communes in Benin, as it would have artificially reduced the demographic stochasticity and underestimated the uncertainty. We thus picked the smallest real population size and applied it to all the simulations. Final results are then rescaled to the real population size. We have clarified it in the text (line 228):

***In order to measure severe cases and deaths which are rare events deaths, we used a simulated population size of*** *50,000 individuals.* ***This was the population size*** *of the least populated commune in 2024, Toucountouna,* ***to avoid simulating a population larger than the actual population (which would have artificially reduced the demographic stochasticity and underestimated the uncertainty), and already resulting in a runtime of up to two hours per individual simulation****. All* ***epidemiological indicators*** *were* ***then*** *rescaled to the real population size*

**- Line 316: the number of averted cases would be the difference between the estimate obtained with an extension and the number given the planned interventions. Hence, should not be I_extension minus I_planned?**

We expect the extension scenario to result in less cases than only the planned interventions (as shown in Figure 5), so to have a non-negative number of averted cases we do substract the cases under the extension scenario from the cases under the planned interventions (line 248 in the revised manuscript).

**- Line 356: MIS and DHS were not defined. Also, the consideration in this sentence should be in the Discussion.**

Thank you for noticing this. We replaced MIS and DHS by “household surveys” line 272, since we use the data from these different surveys in the same way we thought it was not necessary to distinguish their names. We kept the correlation coefficient with the published surveys used to produce the calibration estimates in the Results and moved the rest of the text to a goodness of fit section of the Discussion.

*MAP’s yearly prevalence estimates are based on* ***household*** *surveys which are conducted during a few months and are powered to be representative at the department level [38]. When comparing modelled prevalence in each department during the same months to those surveys, we obtained an adjusted R^2^ of* ***0.79*** *(Figure 3B,* ***2011 and 2017 surveys****).*

***We had to calibrate to global estimates as there were no yearly estimates of prevalence in Benin available. The 2011 and 2017 household surveys [38] were used to produce the prevalence estimates, while the 2015 survey [39] was not and thus also not used for calibration. When including this 2015 survey in the comparison of departmental modelled monthly prevalence to survey prevalence, we obtained an adjusted R^2^ of 0.59.***

**- Line 387: I believe this consideration is for the Discussion.**

Thank you, we have moved this sentence to the new goodness of fit section in the Discussion:

***When validating modelled malaria cases,*** *as expected the number of modelled malaria episodes was substantially higher than both the World Malaria Report and routine data, due to OpenMalaria’s large threshold for clinical case definition and the double counting of illnesses lasting over 30 days [51].*

**- Line 411: the intervals require a different dash character to distinguish negative numbers. Also, the Discussion would benefit from the interpretation of these negative numbers.**

Thank you for this valuable suggestion, the intervals are now written as *3 (-1****,*** *7).*

The negative uncertainty bounds around averted severe cases and deaths are due to the rarity of these events, which makes them more sensitive to stochasticity. This means that in some simulations, the simulated number of deaths with planned interventions can be slightly lower than with one of the extensions. If we increased the simulated population size we would avoid these limit cases, but as explained above we did not want to artificially reduce the stochasticity by simulating a population larger than the real one. We added this sentence to the Discussion:

***Severe cases and deaths are rare events which are sensitive to stochasticity, so averted severe cases and deaths have large uncertainty bounds which can sometimes contain negatives values without corresponding to real events.***

**- Line 447: again better fit in the Discussion.**

We already had a similar statement in the Discussion, and agree that the one in the Results was not necessary. We removed the statement from the Results and slightly edited the one in the Discussion:

***This means*** *the differences* ***in risk*** *we took into account* ***in our model*** *were not strong enough to drive a differentiated impact given the level of model and data uncertainties. Other considerations, such as the cost of extending to each zone, could* ***have been*** *be taken into account to prioritise where to implement SMC first. In the end, the availability of community health workers guided the order of implementation.*

**- Line 495-496: why unit costs obsolete?**

The previous unit costs were taking into account costs associated to training and transporting staff as well as commodities costs for the medicine. At the same time as the SMC extensions scenarios were being considered, the delivery mode of SMC has changed: SMC was delivered through campaigns until 2023, and from 2024 it is delivered by trained community health workers. Therefore, while the unit costs for commodities are still accurate, delivery and training costs were obsolete, and hence, the new overall unit cost is expected to be different from the past one. We clarified it in the text lines 390-394:

***From 2019 to 2023 SMC in Benin was delivered as campaigns, with specific staff trained each year, but since 2024 SMC is administered by community health workers. The previous unit costs, which include commodity but also delivery and training costs, are thus obsolete since the administration of SMC can now rely on pre-existing trained health workers and delivery mechanisms, rather than having to put them in place for each campaign.***

**- Line 504 - 505: I recommend some caution is required as the sentence provides premature statements.**

We have edited the text as follows (line 399):

***Although a detailed costing is beyond the scope of this work****, the difference in impact is such that the demographic extension would have to cost at least four times less per targeted child to avert the same number of severe cases or deaths per dollar spent.*

**- Line 602: I also recommend refraining from talking about “return on investment.”**

We removed this sentence.
